# Clarifying Frizzled 2 function in development through genetically validated mouse models

**DOI:** 10.1242/dmm.052410

**Published:** 2026-06-26

**Authors:** Megan N. Michalski, Cassandra R. Diegel, Zhendong A. Zhong, Maddison E. Marshall, Gabrielle E. Foxa Wiartalla, Payton D. Stevens, Kelly Suino-Powell, Levi L. Blazer, Jarrett J. Adams, Karsten Melcher, Sachdev S. Sidhu, Stephane Angers, Bart O. Williams

**Affiliations:** ^1^ Department of Cell Biology, Van Andel Institute, Grand Rapids, Michigan 49503, USA; ^2^ Department of Structural Biology, Van Andel Institute, Grand Rapids, Michigan 49503, USA; ^3^ Donnelly Centre for Cellular and Biomolecular Research, University of Toronto, Toronto, Ontario M5S 3E1, Canada; ^4^ School of Pharmacy, University of Waterloo, Kitchener, Ontario N2G 1C5, Canada; ^5^ Department of Molecular Genetics, University of Toronto, Toronto, Ontario M5S 3G9, Canada; ^6^ Department of Pharmaceutical Sciences, Leslie Dan Faculty of Pharmacy, University of Toronto, Toronto, Ontario M5S 3M2, Canada; ^7^ Department of Biochemistry, University of Toronto, Toronto, Ontario M5S 1A8, Canada; ^8^ Core Technologies and Services, Van Andel Institute, Grand Rapids, Michigan 49503, USA

**Keywords:** CRISPR/Cas9, Cre-*lox* recombination, Wnt signaling, Frizzled, Fzd, Homologous recombination, Whole-genome sequencing, Limb development, Chondrocytes

## Abstract

Wnt receptors of the Frizzled (Fzd) family are widely considered to exhibit substantial functional redundancy, complicating efforts to therapeutically target individual receptors. Fzd2 was believed to be functionally redundant with Fzd1 and Fzd7, based on previously published global knockout mouse studies. By contrast, homozygosity for a Fzd2 global knockout mouse allele developed by the International Mouse Phenotype Consortium (IMPC) has been reported to cause embryonic lethality, suggesting that Fzd2 is critical for early embryonic development. If global deletion of Fzd2 leads to early lethality, conditional deletion models are necessary to identify tissue-specific phenotypes. We found that a previously published Fzd2 conditional deletion model does not eliminate Fzd2. We have generated a new conditional model to address the contradictory previous studies and allow tissue-specific studies of Fzd2. We successfully inserted two *loxP* sites around the *Fzd2* gene and confirmed that subsequent Cre-mediated recombination creates a *Fzd2* null allele. Global deletion of Fzd2 in this model does not cause embryonic lethality while limb-specific deletion causes limb shortening. This work supports the hypothesis that Fzd2 regulates limb development and emphasizes the importance of thoroughly validating newly generated mouse models.

## Teaser

**Summary:** Previous animal models used to study the role of the Wnt signaling receptor Frizzled 2 (FZD2) had conflicting phenotypes. We developed a new FZD2-conditional-deletion mouse model to definitively determine FZD2 loss-of-function phenotypes.

## INTRODUCTION

WNT signaling pathways regulate embryonic patterning, tissue morphogenesis and skeletal development by coordinating cell proliferation, differentiation and polarity (Nusse and Clevers, 2017; Zhong et al., 2021). These pathways are transduced by Frizzled (FZD) receptors, which can activate both β-catenin-dependent (canonical) and β-catenin-independent (non-canonical) signaling cascades depending on ligand availability, receptor context and intracellular interactions. Among the ten mammalian FZD receptors, FZD2 has emerged as a key regulator of skeletal and craniofacial development (Yu et al., 2010; Zhu et al., 2023), and is directly implicated in human congenital disease (Saal et al., 2015).

Heterozygous pathogenic variants in *FZD2* are associated with the human syndromes autosomal dominant omodysplasia (ADO) (Nagasaki et al., 2018; Saal et al., 2015; Türkmen et al., 2017; Warren et al., 2018) and autosomal dominant Robinow syndrome (ADRS) (White et al., 2018). These related syndromes are rare congenital disorders characterized by limb shortening and craniofacial dysmorphology. ADRS, as well as its recessive form, are caused by mutations in components of the β-catenin-independent WNT pathway, including *WNT5A* (Person et al., 2010), Dishevelled 1-3 (i.e. *DVL1*, *DVL2*, *DVL3*) (White et al., 2015; Bunn et al., 2015) and *ROR2* (Afzal et al., 2000). Given the association of these genes with the planar cell polarity (PCP) pathway, it is presumed that altered PCP signaling is a key contributor to ADRS phenotypes. Human genetic studies have identified multiple classes of ADRS-associated *FZD2* mutations, including truncating alleles affecting the C-terminal DVL interaction domains and missense variants within conserved extracellular or intracellular regions. Together, these findings suggest that disease pathogenesis arises from altered receptor signaling rather than simple haploinsufficiency.

We have previously reported a mouse model that directly links *Fzd2* mutations to ADRS-associated phenotypes (Liegel et al., 2023). In that study, truncating *Fzd2* alleles analogous to those identified in ADO/ADRS patients result in severe craniofacial and limb defects, accompanied by a reduction in the phosphorylation of VANGL2, a key WNT/planar-cell-polarity protein. These findings have established that disruption of the FZD2 C-terminus is sufficient to cause profound developmental abnormalities and demonstrate a causal relationship between *Fzd2* mutations and disease-relevant phenotypes. Subsequently, Zhu and colleagues generated a mouse model carrying a single-nucleotide insertion in the DVL-interacting domain of *Fzd2*, producing a frameshift mutation that disrupts the receptor C-terminus (Zhu et al., 2023). These mutants exhibited shortened limbs, cleft palate, defects in chondrocyte elongation and orientation, and reduced canonical WNT signaling. The study has provided strong evidence that FZD2 regulates skeletal development by mediating both β-catenin-dependent and β-catenin-independent WNT pathways, and further supports the pathogenicity of C-terminal *FZD2* mutations. More recently, Tophkhane et al. (2024) investigated ADRS-associated human *FZD2* missense variants by using avian and cell-based models. All three of these studies studies demonstrate that specific FZD2 variants are sufficient to disrupt craniofacial morphogenesis and chondrogenesis, often through dominant-negative effects on WNT signaling. Importantly, variant-specific differences were observed in canonical and non-canonical pathway outputs, highlighting the complexity of FZD2 signaling and reinforcing the idea that ADRS-associated mutations perturb signaling balance rather than abolishing receptor function outright.

Independent of ADRS-associated alleles, several loss-of-function and knockout models have been used to study a broader role of FZD2 in development. Early germline *Fzd2* knockout studies have reported partially penetrant cleft palate, cardiac septation defects and tissue closure abnormalities, particularly when combined with mutations in related FZD receptors or core PCP components (Yu et al., 2010, 2012). Notably, these phenotypes occur without widespread transcriptional changes, suggesting that FZD2 primarily regulates morphogenetic cell behaviors rather than acting solely through canonical transcriptional programs. In contrast, the International Mouse Phenotyping Consortium (IMPC) has generated an independent global null allele of *Fzd2* and reported early embryonic lethality –i.e. before embryonic day (E) 9.5 – in homozygous *Fzd2-*null animals (Dickinson et al., 2016). Tissue-specific deletion studies further implicated FZD2 in non-canonical WNT-dependent processes (Kadzik et al., 2014). Moreover, conditional disruption of *Fzd2* in epithelial or mesenchymal compartments alters tissue architecture without consistently reducing β-catenin-dependent WNT target gene expression.

Despite these advances, interpretation of existing *Fzd2* mouse models is complicated owing to several technical limitations. The original conditional *Fzd2* allele was intended to function as a conventional floxed allele (Kadzik et al., 2014). However, we found that this allele harbors a complex genomic rearrangement, including an inverted duplication of the *Fzd2* locus with oppositely oriented *loxP* sites (Michalski et al., 2021 preprint). As a consequence, Cre-mediated recombination of this allele results in continuous inversion and production of antisense transcripts rather than gene deletion. This structural configuration has been independently validated and formally reported (Zhu et al., 2023). As a result, phenotypes observed using this allele reflect combined effects of altered gene dosage and antisense RNA-mediated suppression of gene expression rather than isolated loss of *Fzd2* function. Additional ambiguity arises from the original germline *Fzd2* knockout allele, which retains a portion of the C-terminal coding sequence and may produce truncated protein products with residual signaling capacity. Finally, phenotypes reported for the IMPC *Fzd2* allele are not fully consistent with those observed in other *Fzd2* mutant models, particularly with respect to skeletal outcomes and early embryonic lethality. Collectively, these issues underscore the need for a rigorously validated conditional *Fzd2* allele that enables unambiguous assessment of FZD2 function in specific tissues and developmental contexts.

To address the limitations of existing *Fzd2* mouse models, we pursued two complementary genetic strategies. First, we generated a revised Fzd2 knockout allele that eliminates the residual C-terminal coding sequence retained in the original germline knockout (Yu et al., 2010), thereby producing a *Fzd2* null allele without the potential for truncated protein products or residual signaling activity. This approach enables direct reassessment of phenotypes previously attributed to *Fzd2* loss of function and clarifies the contribution of the FZD2 C-terminus to signaling. Second, we generated a conditional *Fzd2* allele that allows precise, Cre-dependent inactivation of *Fzd2* while preserving normal gene structure and regulation prior to recombination. By using these genetically rigorous alleles, we define the consequences of complete and tissue-specific *Fzd2* ablation *in vivo*, and clarify how FZD2 regulates limb morphogenesis and WNT pathway output. Our findings reconcile discrepancies among prior *Fzd2* models and establish a robust genetic framework for investigating how FZD2 contributes to development and human skeletal disease.

## RESULTS

### Previously published Fzd2 mouse models do not produce complete null alleles

We obtained two previously published Fzd2 mouse models, one comprising the *Fzd2^tm1.1Nat^* allele (Yu et al., 2010) and has been reported to yield a global knockout, and one comprising the *Fzd2^tm1Eem^* allele (Kadzik et al., 2014) that was intended to be a conditional allele. Mice homozygous for the *Fzd2^tm1.1Nat^* allele do not express N-terminus FZD2 protein; however, we noticed that the *Fzd2^tm1.1Nat^* allele retains a portion of the CDS (531 bp) that could retain partial function (Fig. S1A). Flow cytometric analysis confirmed deletion of the N-terminus of FZD2 (Fig. S1B). However, we detected mRNA expression of the retained *Fzd2* C-terminal fragment in E11.5 limb lysates isolated from *Fzd2^tm1.1Nat^* homozygotes (Fig. S1C). There are several potential alternative start codons within the remaining sequence, including an in-frame ATG with a strong Kozak sequence (Kozak, 1987) near the 5′ end of this Fzd2 fragment (a 531 bp in-frame CDS is predicted, Fig. S1A). Because there are no antibodies available that recognize the C-terminal region of FZD2, we expressed a C-terminal epitope-tagged (1D4) version of this *Fzd2* fragment (Fzd2-delta-N), which initiates transcription from this ATG in HEK293 cells (Fig. S1D). This fragment localized in a manner similar to that of full-length FZD2 (Fig. S1D,E) and can activate the β-catenin-responsive TOPFlash reporter in HEK293 cells in response to WNT3A and RSPO1 (de Lau et al., 2014) (Fig. S1F). Fzd2-delta-N activity depends on endogenous FZDs as it has no Wnt signaling activity in HEK293T cells that lack all ten FZDs (FZDless cells) (Fig. S1F) (Eubelen et al., 2018). One possibility to explain the signaling capacity of the C-terminal fragment is that the carboxyl portion of FZD2 can partner with at least one wild-type Fzd family member to transduce canonical Wnt signaling. Another possibility is that FZD-delta-N can interact with other endogenous FZD-regulating proteins to affect other FZDs. These *in vitro* studies collectively support the hypothesis that the *Fzd2^tm1.1Nat^* allele is hypomorphic. By using the improved genome-editing via oviductal nucleic acids delivery (i-GONAD) method (Ohtsuka et al., 2018; Gurumurthy et al., 2019), we applied a two-guide-RNA CRISPR/Cas9 strategy to delete the remaining FZD2 CDS in *Fzd2^tm1.1Nat^* (Fig. S1G) animals. We used PCR-based genotyping and Sanger sequencing to confirm the *Fzd2^KO-delta-Cterm^* allele was correctly generated. We noticed the presence of cleft palate in ∼53% of animals homozygous for the *Fzd2^KO-delta-Cterm^* allele (Fig. S1H).

We also obtained a previously reported *Fzd2^tm1Eem^* flox strain (Kadzik et al., 2014) but determined that complete deletion of Fzd2 does not occur after exposure to Cre. Whole-genome sequencing (WGS) of mice homozygous for *Fzd2^tm1Eem^* flox confirmed that the allele is a complex genomic alteration comprising a *Fzd2* gene duplication (Fig. S2A,F-H). The *Fzd2^tm1Eem^* flox allele contains two copies of Fzd2, separated by insertion of a large 10-kb exogenous DNA sequence, in addition to a region (Fig. S2A, black curved line) that is too long to be sequenced as a single continuous read. Additionally, we noticed a 100 kb endogenous sequence downstream of the *Fzd2* gene, which is duplicated and likely inverted (Fig. S2A,F).

One region of the Fzd2 coding DNA sequence (CDS) is within the sequence flanked by *loxP* sites that, we believe, are inserted in opposite orientations. We hypothesize that this leads to the sequence inverting between the *loxP* sites upon Cre-mediated recombination, resulting in the two Fzd2 copies being in tandem (Fig. S2A, see Mutant). Consistent with the duplication seen after WGS, genomic Fzd2 DNA copy numbers were increased in homozygous flox mice (Fig. S2B,C,G). Germline Cre-mediated recombination only mildly reduced FZD2 protein and *Fzd2* mRNA expression in limb buds (Fig. S2D,E).

Qualitative phenotypic assessment of *Fzd2^tm1Eem^* revealed several strong phenotypes. Consistent with the findings described by Zhu et al. (2023), mice heterozygous for both Prx1-Cre and the *Fzd2^tm1Eem^* alleles have short limbs (Fig. S3A). Notably, we were unable to produce mice heterozygous for *Prx1-Cre* and homozygous for the *Fzd2^tm1Eem^* allele. Deletion of the *Fzd2^tm1Eem^* allele in osteoblasts through *OC-Cre* resulted in reduced trabecular bone patterning and narrowing of the cortical bone in adult mice (Fig. S3B).

Craniofacial skeletal abnormalities were observed by micro-computed tomography (microCT) analysis (Fig. S3C-E), including shortening of the face and decreased mineralized tissue. In disease models, Cre-mediated recombination of the *Fzd2^tm1Eem^* allele in pancreatic epithelial cells altered survival, with accelerated disease progression relative to standard *Kras^G12D/+^;Pdx1-Cre^Tg/+^;Fzd2^+/+^* (KC) and *Kras^G12D/+^;P53^R172H/+^;Pdx1-Cre^Tg/+^;Fzd2^+/+^* (KPC) cohorts (Fig. S3F). These findings are consistent with the presence of an unintended hypomorphic or neomorphic allele and underscore that phenotypes observed when using the *Fzd2^tm1Eem^* allele do not accurately reflect conditional Fzd2 loss.

### Generation and validation of a new conditional Fzd2 allele

**Fig. 1.**
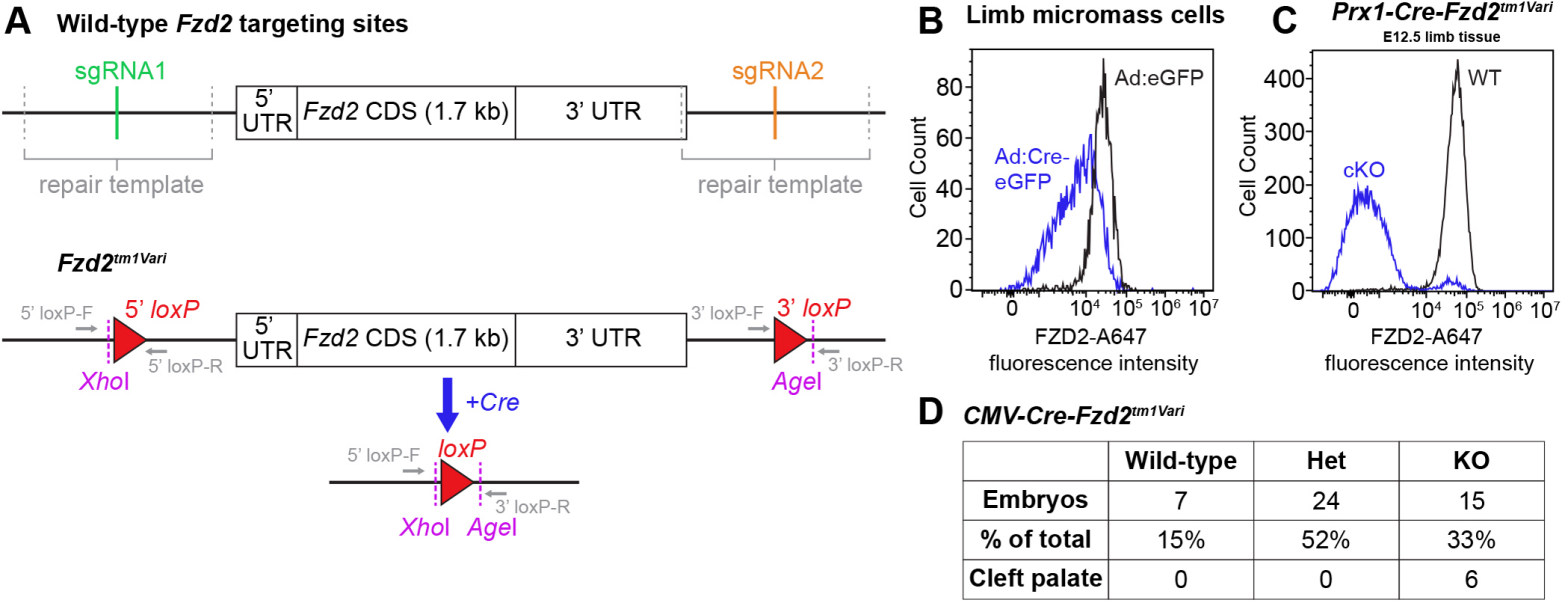
**Generation and confirmation of the *Fzd2^em1Vari^* allele.** (A) Top: Schematic detailing the location of guide RNAs (indicated by the green and orange text on map) and repair template regions of homology. Bottom: Schematic detailing the B6;C3H-*Fzd2^em1Vari^* intended modifications. *LoxP* sites were placed around the entire single exon *Fzd2* gene. *Xho*1 and *Age*1 restriction sites (purple) were incorporated to validate the allele. Red triangles indicate *LoxP* sites; 5′ and 3′ untranslated regions (UTRs) are indicated. Primer binding sites for genotyping are shown in gray. (B) Flow cytometric histograms showing FZD2 (Alexa Fluor 647) fluorescence intensity (logarithmic scale) in live eGFP-positive cells from *Fzd2*^*em1Vari*^ homozygous micromass cultures infected with control adenovirus (Ad:eGFP) or Cre-expressing adenovirus (Ad:Cre-eGFP). Cells were gated to exclude debris and doublets, live cells were identified as DAPI-negative, and eGFP-positive cells were analyzed. Reduced FZD2-A647 fluorescence following Ad:Cre-eGFP infection confirms Cre-mediated deletion of Fzd2. (C) Flow cytometric histograms showing FZD2-A647 fluorescence intensity (logarithmic scale) in live single-cell suspensions prepared from E12.5 limbs from *Prx1-Cre;Fzd2*^*em1Vari*^ +/+; fl/fl wild-type (WT) and Tg/+;fl/fl conditional knockout (cKO) animals. Cells were gated to exclude debris and doublets and live cells were identified as DAPI-negative. Reduced FZD2-A647 fluorescence in cKO cells confirms deletion of FZD2 protein in limb mesenchyme. (D) Genotype distribution and cleft palate incidence among E17.5–E18.5 *CMV-Cre-Fzd2^em1Vari^* offspring from heterozygous (HET)×heterozygous matings (*n*=46). Six KO embryos (*n*=15 in total) had a cleft palate.

Due to the complex issues noticed with the *Fzd2^tm1Eem^* flox allele, we generated another conditional allele *Fzd2^em1Vari^* by using a modified (2C-HR)-CRISPR approach (Gu et al., 2018). To increase editing efficiency, we cloned, expressed and purified the Cas9-streptavidin (Cas9-mSA) fusion protein (Fig. S4A). We confirmed that the addition of the streptomycin tag did not affect the ability of the modified Cas9 to cut linearized DNA (Fig. S4B). To avoid altering endogenous *Fzd2* expression, the 5′ *loxP* site was inserted into a region upstream (5′) of the promoter (Fig. 1A), with the 3′ *loxP* site being 994 bp downstream of the 3′ UTR (Fig. 1A). Of the 85 live-born animals, four carried both the 5′ and 3′ *loxP* insertions. Germline transmission and *cis*-integration of both sites were confirmed by PCR-based genotyping and Sanger sequencing (Fig. S4C,D). Correct *loxP* insertion and orientation were further validated by restriction digest analysis and WGS, which confirmed the absence of unintended genomic rearrangements or duplications (Fig. S4E). These data demonstrate successful generation of a structurally intact and precisely engineered Fzd2 conditional allele.

To assess Cre-mediated recombination of *loxP* sites (Fig. 1A) *in vitro*, we used micromass culture technique to analyze limb bud cells. Limb micromass cultures from wild-type or *Fzd2^em1Vari^* homozygous flox animals were infected with adenovirus expressing Cre (Ad5CMV:Cre-eGFP) or control adenovirus (Ad5CMV:eGFP). Infection was confirmed by fluorescence imaging (Fig. S4F). Genotyping by PCR amplification confirmed that a product was present at the anticipated size after Cre-mediated recombination (Fig. S4G). Sanger sequencing confirmed Cre-mediated recombination resulted in the deletion of the Fzd2 coding sequence between the *loxP* sites, retaining sequence for one *loxP* site and *Xho*I and *Age*I restriction sites. Cell suspensions were stained using an antibody against FZD2 and analyzed by flow cytometry. *Fzd2^em1Vari^* homozygous flox micromass cultures infected with Ad5CMV:Cre-eGFP showed a reduction in FZD2 expression compared to control, confirming Cre-mediated deletion decreased FZD2 protein (Fig. 1B).

To assess the role of Fzd2 specifically in limb development while avoiding possible confounding effects of early lethality, we generated the *Prx1-Cre-Fzd2^em1Vari^* strain to delete Fzd2 in limb mesenchyme. *Prx1-Cre-Fzd2^em1Vari^* limb (E11.5) tissue cell suspensions were analyzed by flow cytometry for FZD2 membrane protein expression. *Fzd2^em1Vari^* homozygous knockout limbs showed a dramatic reduction in FZD2 expression compared to wild-type littermate controls, confirming FZD2 deletion (Fig. 1C). To confirm *in vivo* Cre-mediated recombination, we crossed female mice homozygous for CMV-cre (Tg/Tg) with male mice homozygous for the *Fzd2^em1Vari^* allele. CMV-cre was used to delete *Fzd2* in all tissues, including germ cells. Offspring were backcrossed to C57BL/6J animals to confirm germline transmission. Heterozygous (KO/+) offspring from this second cross were then mated to generate Fzd2 homozygous knockouts. Embryos were collected at E17.5–E18.5. Homozygous knockout animals could be detected at E17.5–E18.5 and appeared grossly normal, albeit with partially penetrant cleft palate (Fig. 1D) consistent with the finding from the *Fzd2^tm1.1Nat^* allele (Yu et al., 2010).

### Limb elements are shortened in embryonic and adult Fzd2 loss-of-function models

**Fig. 2.**
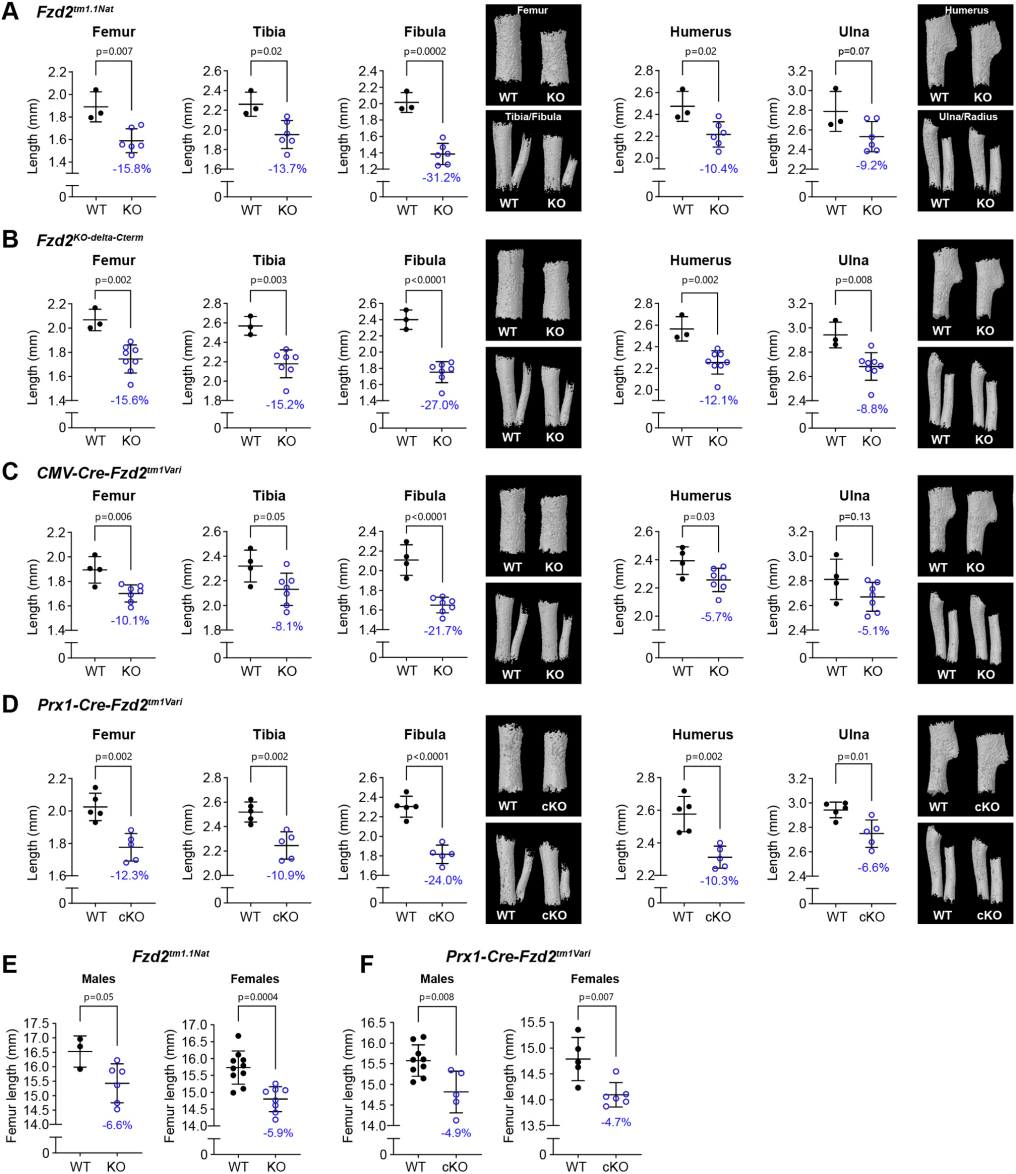
**Limb elements of Fzd2 null allele mice at E18.5 and adult stage are shorter than those of wild-type mice.** (A-D) Limbs from *Fzd2^tm1.1Nat^*, *Fzd2^KO-delta-Cterm^*, *CMV-Cre-Fzd2^em1Vari^* and *Prx1-Cre-Fzd2^em1Vari^* animals at E18.5 were collected, fixed and then scanned for micro-computed tomography (microCT) analysis. Fore- and hindlimb length was measured. Percent changes relative to wild-type (WT) controls are reported under knockout (KO) and conditional knockout (cKO) values. All Fzd2 loss-of-function models exhibited shortened limb elements compared with WT controls. For each animal group representative 3D images of limb elements are shown. Individual data points indicate the mean±standard deviation (s.d.); *P*-values are indicated on each plot. (E,F) Femurs from 3-month-old *Fzd2^tm1.1Nat^* and *Prx1-Cre-Fzd2^em1Vari^* animals were collected, fixed and scanned for microCT analysis. Bone lengths were measured. Fzd2 loss-of-function models exhibited shortened femurs compared with WT controls. Individual data points indicate the mean±s.d.; *P*-values are indicated on each plot; *P*<0.05 was considered significant.

Analysis of E18.5 embryos revealed consistent shortening of forelimb and hindlimb elements across all Fzd2 loss-of-function models examined, including *Fzd2^tm1.1Nat^, Fzd2^KO-delta-Cterm^, CMV-Cre-Fzd2^em1Vari^* and *Prx1-Cre-Fzd2^em1Vari^* (Fig. 2A-D). Deletion of the residual C-terminal coding sequence in the original *Fzd2^tm1.1Nat^* allele did not exacerbate limb shortening, indicating that this retained fragment is not responsible for the observed skeletal phenotype (Fig. 2A,B). Similarly, both global and limb mesenchyme-specific deletion of Fzd2 by using the newly generated *Fzd2^em1Vari^* allele resulted in comparable reductions in limb length (Fig. 2C,D).

These defects persisted into adulthood. Femurs from 3-month-old *Fzd2^tm1.1Nat^* and *Prx1-Cre-Fzd2^em1Vari^* mice remained significantly shorter than those of control mice of either sex (Fig. 2E,F). Despite shortened bones, trabecular and cortical bone parameters were largely preserved, with only modest genotype- and sex-specific differences detected (Tables S1,S2). Consistent with these findings, calvarial osteoblasts derived from *Fzd2^em1Vari^* mice showed normal mineralization following Cre-mediated deletion *in vitro* (Fig. S5).

### Loss of Fzd2 results in subtle craniofacial skeletal alterations

**Fig. 3.**
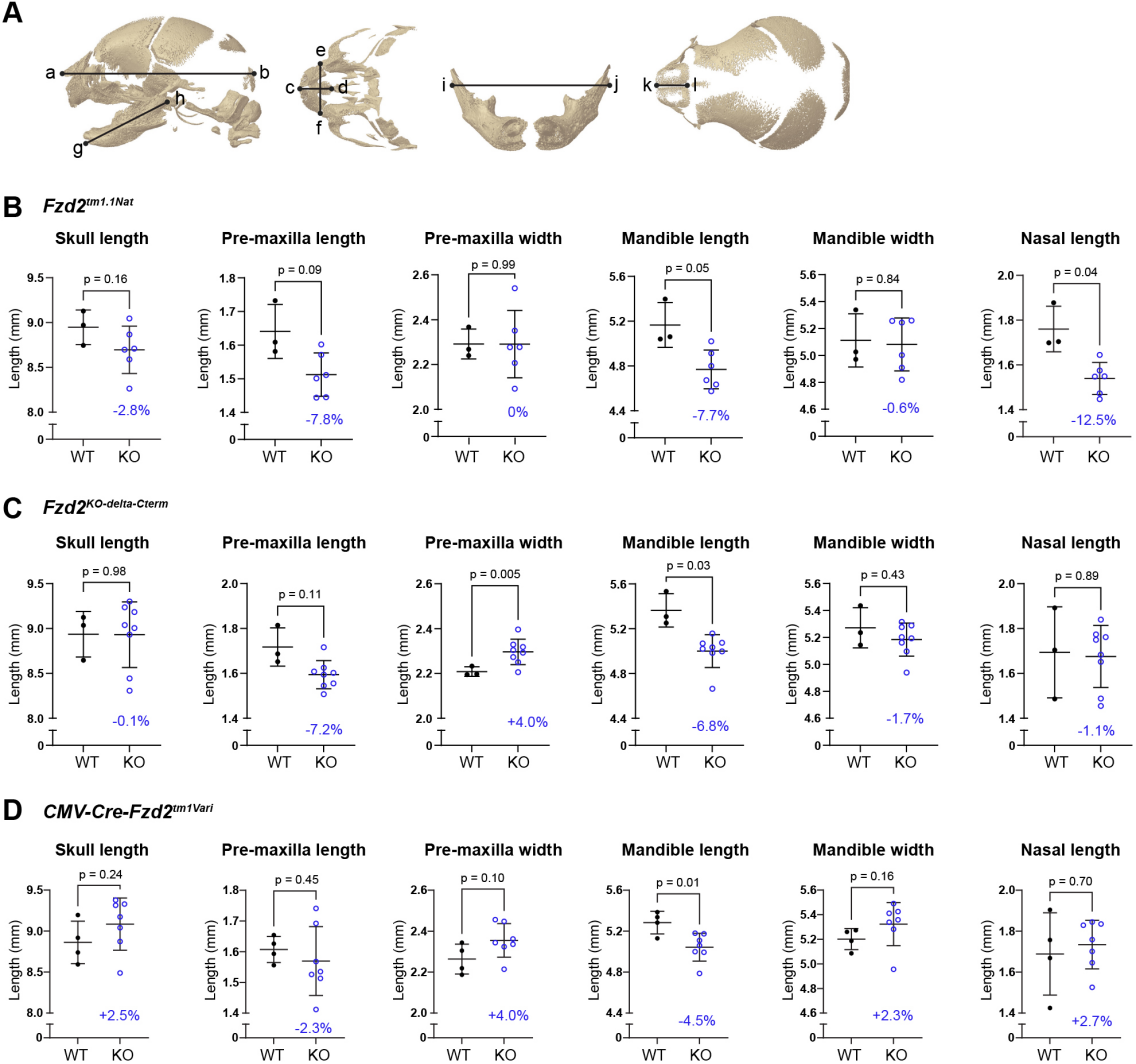
**Quantitative craniofacial analysis of Fzd2 loss-of-function mouse models.** (A) Representative microCT reconstructions of adult mouse skulls showing lateral, ventral, frontal and dorsal views with annotated landmarks used for morphometric measurements. Skull length was measured from a to b (anterior-most point of nasal bone to occipital bone). Pre-maxilla length was measured from c to d (anterior-most to posterior-most point of pre-maxilla bone). Pre-maxilla width was measured from e to f (the lateral-most points of pre-maxilla bone). Mandibular length was measured from g to h (inferior-most point on incisor alveolar rim to posterior-most point on mandibular condyle). Mandibular width was measured from i to j (lateral-most points of left and right mandibular bones). Nasal bone length was measured from k to l (anterior-most to posterior-most point of the nasal bone). (B-D) Quantification of craniofacial dimensions in three independent Fzd2 models: *Fzd2^tm1.1Nat^* (B), *Fzd2^KO-delta-Cterm^* (C), and *CMV-Cre-Fzd2^em1Vari^* (D). Individual data points indicate the mean±standard deviation. Percent change relative to wild-type controls is indicated below each comparison. Mandibular length was consistently reduced in Fzd2 loss of function models compared to WT controls, whereas other craniofacial measurements showed modest and variable effects. Statistical significance was assessed using unpaired two-tailed Student's *t*-tests; *P*-values are shown above each comparison; *P*<0.05 was considered significant.

Quantitative craniofacial measurements revealed modest changes in select parameters across Fzd2 loss-of-function models (Fig. 3A-D). Mandible length was consistently reduced in all three genotypes examined, whereas other measurements, including skull length, pre-maxilla width and nasal length, showed smaller and more variable effects. Notably, no genotype displayed the pronounced midface hypoplasia or facial widening characteristic of ADRS. These data indicate that complete Fzd2 deletion results in subtle craniofacial alterations rather than overt syndromic craniofacial dysmorphology.

### Canonical Wnt target gene expression is preserved following Fzd2 deletion

**Fig. 4.**
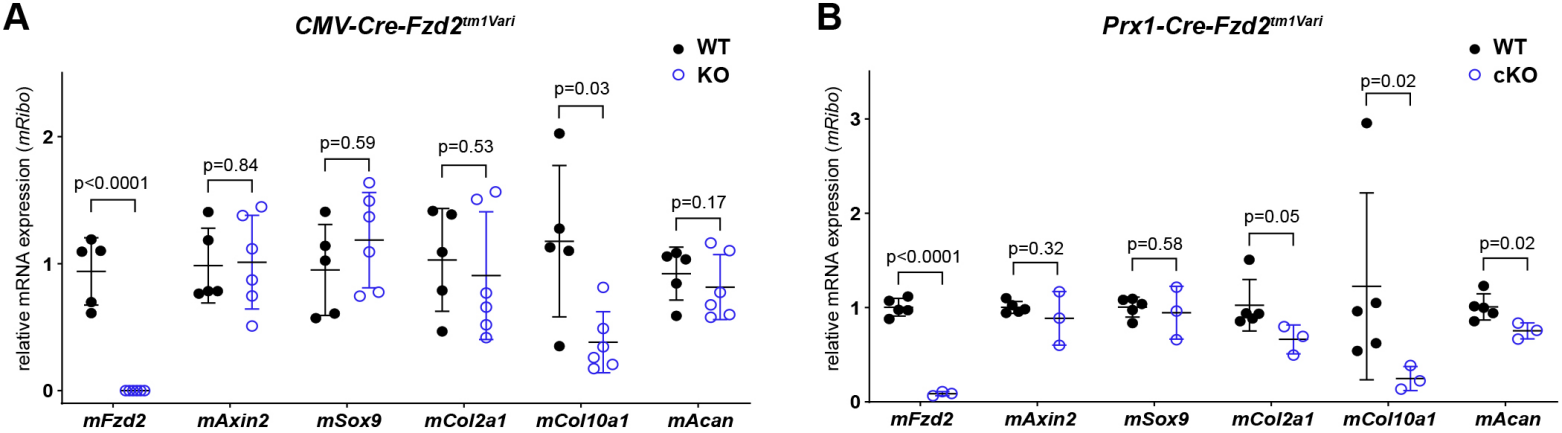
***Col10a1* expression is reduced in the limbs of Fzd2-deficient mice at E14.5.** (A,B) Quantitative RT-PCR analysis. Plotted is the relative mRNA expression of *mFzd2*, *mAxin2*, *mSox9*, *mCol2a1*, *mCol10a1* and *mAcan* in limb lysates of *CMV-Cre-Fzd2^em1Vari^* (A) and *Prx1-Cre-Fzd2^em1Vari^* (B) embryos at E14.5. mRNA expression levels were normalized to those of ribosomal RNA (*mRibo*) and are shown relative to wild-type (WT) controls. *Fzd2* and *Col10a1* expression was reduced in Fzd2 knockout tissues relative to those of WT control, whereas expression of *Axin2*, Sox9, *Col2a1* and *Acan* was largely unchanged. Individual data points indicate the mean±standard deviation. *P*-values are indicated above comparisons; *P*<0.05 was considered significant.

To determine whether Fzd2 deletion alters β-catenin-dependent WNT signaling *in vivo*, we measured expression of the canonical WNT target gene *Axin2* in limb lysates from three independent Fzd2 loss-of-function models, i.e. *Fzd2^tm1.1Nat^*, *CMV-Cre-Fzd2^em1Vari^* and *Prx1-Cre-Fzd2^em1Vari^*. *Axin2* expression was not significantly altered in any model compared to genotype-matched controls, indicating that canonical WNT/β-catenin signaling is largely preserved following Fzd2 loss (Fig. 4A,B; Fig. S6A-C). As expected, *Fzd2* mRNA was significantly reduced in all mutant contexts, confirming effective gene deletion (Fig. 4A,B; Fig. S6A-D). Consistent with prior reports (Yu et al., 2010), mRNA expression of other *Fzd* receptors (*Fzd1–Fzd10*) was not detectably altered in *Prx1-Cre-Fzd2^em1Vari^* limbs, suggesting limited transcriptional compensation at the receptor level (Fig. S6D).

### Stage-specific changes in chondrocyte maturation markers at E14.5

Because limb shortening could be triggered by alterations in chondrocyte differentiation or maturation, we measured the expression of key chondrogenesis markers, i.e. *Sox9*, *Col2a1*, *Col10a1* and *Acan*. Expression of *Sox9*, a master regulator of early chondrocyte specification, was unchanged across all genotypes examined and at multiple developmental time points (Fig. 4A,B; Fig. S6A-C). In addition, expression of *Col2a1* and *Acan*, which marks chondrocytes differentiation and cartilage matrix production, was not significantly altered in limb lysates of Fzd2 null animals at E12.5 (Fig. S6A-C). *Col10a1* expression was unchanged at E12.5 across all genotypes (Fig. S6A-C). However, a significant reduction in *Col10a1* expression was observed at E14.5 in Fzd2-deficient models, including *CMV-Cre-Fzd2^em1Vari^* (Fig. 4A) and *Prx1-Cre-Fzd2^em1Vari^* (Fig. 4B), indicating a stage-specific effect on a marker of hypertrophic chondrocytes. Together, these data indicated that bulk transcriptional programs associated with early chondrocyte differentiation appear largely preserved at E12.5. However, the consistent reduction in *Col10a1* expression at E14.5 across models suggested a modest, stage-specific alteration in markers associated with chondrocyte maturation. We note that these analyses were performed on whole-limb lysates and, therefore, do not detect spatially restricted or cell-type-specific differences.

### Fzd2 loss causes context-dependent defects in chondrocyte orientation

**Fig. 5.**
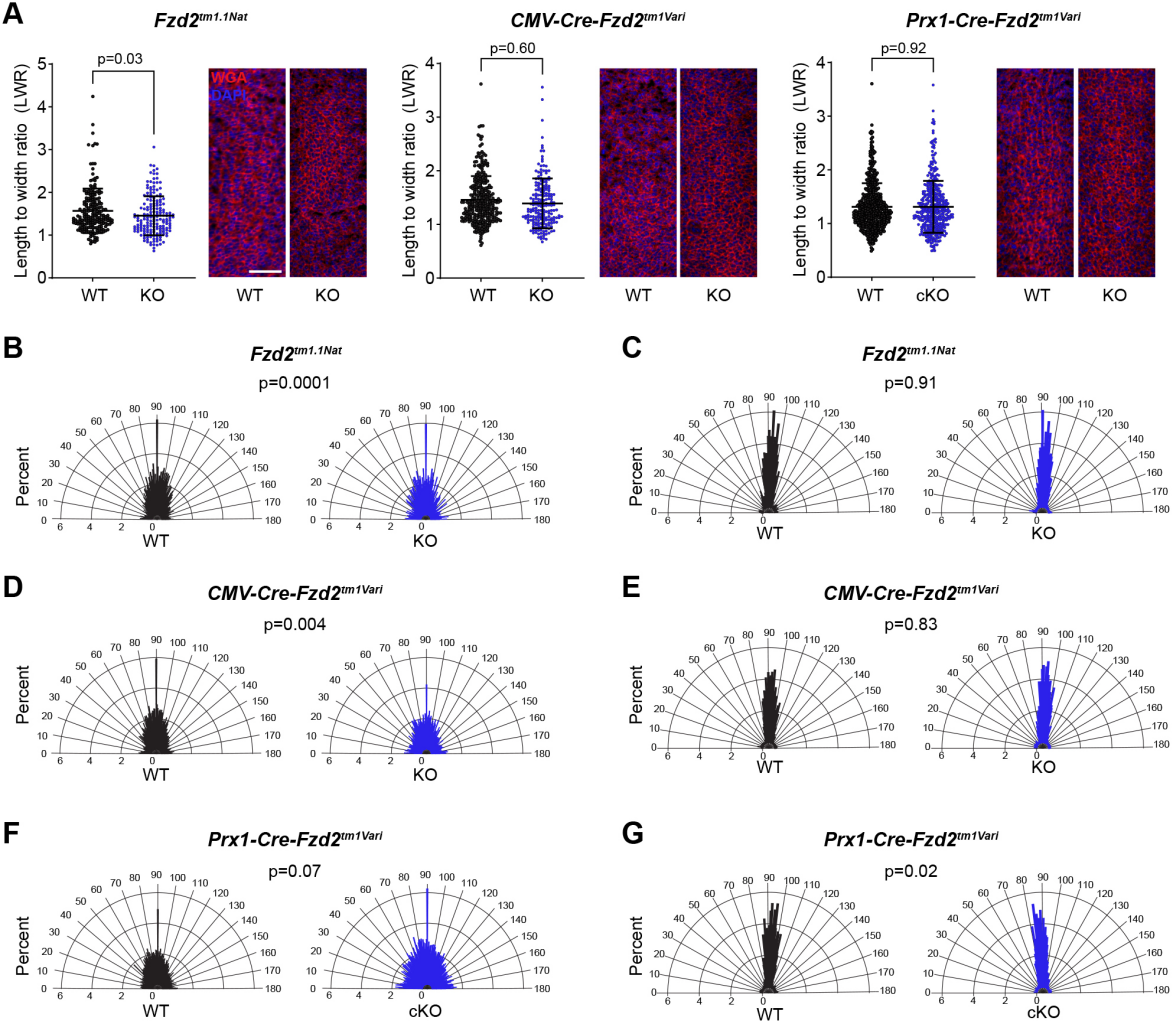
**Fzd2 loss alters chondrocyte orientation during limb development.** (A) Length-to-width ratio (LWR) measurements of chondrocytes within the digits of paws from mice of indicated genotypes at E12.5, plotted as individual measurements with statistical comparisons between wild-type (WT) and knockout (KO) or conditional knockout (cKO) samples. Representative images are shown, scale bar=50 μm. (B-G) Polar histograms showing the distribution of cell orientation angles in developing limb tissues. Cell orientation was quantified in digits at E12.5 (B,D,F) and femur chondrocytes at E18.5 (C,E,G) from *Fzd2^tm1.1Nat^*, *CMV-Cre-Fzd2^em1Vari^* and *Prx1-Cre-Fzd2^em1Vari^* embryos. Percent frequency is plotted as the function of angle relative to the proximal–distal axis. Fzd2 loss had little effect on chondrocyte elongation but caused context-dependent alterations in chondrocyte orientation, consistent with disruption of planar cell polarity. Statistical comparisons of orientation distributions are indicated by *P*-values; *P*<0.05 was considered significant.

To determine whether Fzd2 loss affects non-canonical WNT/PCP-dependent cellular organization, we quantified chondrocyte morphology and orientation in developing limbs. Measurement of cell length-to-width ratios revealed no consistent differences between digits of paws from KO samples of *Fzd2^tm1.1Nat^, CMV-Cre-Fzd2^em1Vari^* or *Prx1-Cre-Fzd2^em1Vari^* mice compared to those from WT mice, indicating that Fzd2 deletion does not grossly alter chondrocyte elongation (Fig. 5A).

In contrast, analysis of cell orientation revealed significant stage- and tissue-specific disruption. Chondrocytes within the digits of *Fzd2^tm1.1Nat^* and *CMV-Cre-Fzd2^em1Vari^* embryos at E12.5 exhibited increased angular dispersion relative to controls, consistent with impaired PCP (Fig. 5B,D). A similar trend was observed in the digits of *Prx1-Cre-Fzd2^em1Vari^* animals, although this was statistically insignificant (Fig. 5F). At E18.5, *Prx1-Cre-Fzd2^em1Vari^* embryos showed altered chondrocyte orientation in femurs, whereas global knockout models did not show persistent polarity defects at this later embryonic stage (Fig. 5C,E,G). These findings indicate that Fzd2 contributed to proper cellular organization during limb development in a context-dependent manner.

### Abundance of Wnt pathway proteins and phosphorylation of VANGL2 are not grossly altered by Fzd2 loss

To determine whether the observed polarity defects were associated with changes in Wnt pathway signaling, we analyzed the abundance of proteins and their phosphorylation states *in vitro* and *in vivo*. Mouse embryonic fibroblasts (MEFs) obtained from *CMV-Cre-Fzd2^em1Vari^* animals were treated with control medium or WNT3A- or WNT5A-conditioned media to stimulate canonical or non-canonical WNT signaling. Western blot analyses revealed robust induction of canonical signaling markers, i.e. of activated β-catenin (CTNNB1, hereafter referred to as ABC) and phosphorylated LDL receptor related protein 6 (pLRP6) in WT as well as Fzd2-deficient cells (Fig. S7A,B). Levels of non-canonical pathway components, including ROR2 and phosphorylated VANGL2 (pVANGL2), were comparable between genotypes and Wnt-induced levels of pVANGL2 were not detectably reduced upon loss of Fzd2 (Fig. S7A,B). In addition, levels of cleaved caspase-3 remained low and variable, indicating no overt increase in apoptosis under these conditions.

Analysis of limb lysates from *Fzd2^tm1.1Nat^* and *CMV-Cre-Fzd2^em1Vari^* embryos at E12.5 similarly revealed comparable levels of ABC, pLRP6, DVL3, ROR1/2 and pVANGL2 between genotypes (Fig. S8A,B). Quantification did not identify significant genotype-dependent differences in these signaling readouts. Together, these data indicate that Fzd2 loss did not cause major changes in the abundance of steady-state canonical or non-canonical Wnt signaling proteins, suggesting that Fzd2-dependent limb phenotypes are triggered by more subtle spatially restricted or context-specific regulation of cell behavior rather than global disruption of Wnt pathway activation.

## DISCUSSION

Wnt signaling is essential for organ development and tissue homeostasis, and its dysregulation contributes to a wide range of human diseases, including osteoporosis, myocardial infarction and cancer (Nusse and Clevers, 2017). The pathway comprises 19 WNT ligands (Mikels and Nusse, 2006) that – together with numerous co-receptors (Maurice and Angers, 2025) – signal via ten FZD receptors (Wang et al., 2016), thereby necessitating a high degree of therapeutic specificity to avoid unintended and potentially debilitating side effects. This challenge is exacerbated by the ability of Wnt ligands to activate several downstream signaling pathways, including the β-catenin (canonical) pathway (Nusse and Clevers, 2017; Daulat and Borg, 2017) and β-catenin-independent (non-canonical) pathways (Veeman et al., 2003; Romereim and Dudley, 2011). Consistent with this complexity, clinical efforts to therapeutically target Wnt signaling have underscored the difficulty of achieving pathway- and context-specific modulation. For example, phase-1 clinical trials assessing inhibitors of porcupine O-acyltransferase porcupine (PORCN), which broadly suppress Wnt ligand secretion, and of Vantictumab, which blocks signaling via five of the ten FZD receptors, were halted due to severe osteoporosis as an unintended side effect (Mirabelli et al., 2019). These outcomes highlight the need to understand the distinct physiological functions of individual Wnt pathway components to develop therapies that minimize harmful side effects.

Studying FZD2 provides a valuable opportunity to dissect receptor-specific functions within the Wnt-signaling network. Previous studies that have used knockout mouse models suggest that, compared with Fzd1 and Fzd7, Fzd2 has highly redundant physiological functions (Yu et al., 2010, 2012). Mice homozygous for the *Fzd2^tm1.1Nat^* allele were viable at birth, although ∼50% died neonatally due to cleft palate. More-severe phenotypes were observed in compound mutants: embryos lacking both Fzd2 and Fzd7 died before gastrulation, while combined loss of both Fzd1 and Fzd2 resulted in fully penetrant cleft palate. Notably, these conclusions of functional redundancy were based on the *Fzd2^tm1.1Nat^* allele, which retains a portion of the C-terminal coding sequence. Overexpression of a truncated Fzd2 fragment mimicking the *Fzd2^tm1.1Nat^* allele revealed residual signaling activity *in vitro*, raising the possibility that this allele is hypomorphic rather than a true null allele. In contrast, the IMPC has generated a Fzd2 null allele that deletes the entire coding sequence and is embryonic lethal prior to E9.5 (Dickinson et al., 2016). This suggests that completed loss of Fzd2 may cause early embryonic lethality, making it impossible to determine specific functions of Fzd2 in any tissues formed later than E9.5 when using a global knockout animal.

To address these inconsistencies, we used the i-GONAD method to remove the retained C-terminus from the *Fzd2^tm1.1Nat^* allele. Mice homozygous for the *Fzd2^KO-delta-Cterm^* allele were phenotypically indistinguishable from animals homozygous for the *Fzd2^tm1.1Nat^* allele, suggesting that – while the C-terminus may have retained some signaling function in *in vitro* over expression systems – it does not account for the phenotypic discrepancies between the *Fzd2^tm1.1Nat^* and the IMPC model.

We also found that a previously published Fzd2 conditional deletion model (Kadzik et al., 2014) harbors a complex genomic duplication that prevents complete gene deletion following Cre-mediated recombination. These structural features likely account for the wide range of phenotypes reported in previous studies, ranging from apparent functional redundancy to early embryonic lethality (Kadzik et al., 2014; Brennan-Crispi et al., 2022; Zhu et al., 2023). In contrast, our *Fzd2^em1Vari^* allele enables unambiguous Cre-mediated deletion of the entire Fzd2 locus without altering gene structure prior to recombination, thereby providing a definitive framework for assessing Fzd2 loss-of-function phenotypes.

When designing the new *Fzd2^em1Vari^* allele, we considered that Fzd2 is a single exon gene located entirely within a CpG island. Therefore, we inserted *loxP* sites fully outside the *Fzd2* locus while minimizing deletion of surrounding genomic DNA to avoid unintended effects on the regulation of neighboring genes following Cre-mediated deletion. To efficiently generate this conditional allele, we adapted a two-cell (2C) homologous recombination (HR) CRISPR strategy (Gu et al., 2018). This method uses mRNA that directs the expression of a Cas9-streptavidin fusion protein in combination with biotinylated PCR templates to enhance homology-redirected repair and enable precise insertion of large genomic modifications. To further streamline this procedure, we produced recombinant streptavidin-tagged Cas (Cas9-mSA) protein for injection. We also assembled ribonucleoprotein (RNP) complexes containing the Cas9-mSA single guide (sg) RNA and biotinylated DNA repair template for *loxP* site to increase efficiency and enable simultaneous targeting of both sites. Following founder identification and backcrossing, we rigorously validated the resulting allele using Sanger sequencing, restriction digestions and WGS. After confirming the allele was a *bona fide* conditional model, we proceeded with phenotypic characterization.

Despite the strong association of heterozygous FZD2 mutations with ADO/ADRS (White et al., 2018), complete deletion of Fzd2 in mice produced only modest craniofacial phenotypes and did not phenocopy the severe facial dysmorphology observed in patients. This distinction is biologically meaningful. Human ADRS-associated alleles are heterozygous and often truncate or alter the FZD2 C-terminus, resulting in dominant-negative or neomorphic effects that perturb signaling balance rather than eliminate receptor function. In contrast, the biallelic loss-of-function models examined here remove Fzd2 entirely and, therefore, do not reproduce the signaling imbalance imposed by mutant receptors. These findings support the conclusion that ADRS arises from altered receptor signaling properties rather than simple FZD2 haploinsufficiency.

Across multiple independent Fzd2 loss-of-function models, we found neither significant reduction in the expression of canonical Wnt target genes, including *Axin2*, nor consistent changes in β-catenin protein levels. These results align with prior reports suggesting that FZD2 is not uniquely required for canonical Wnt transcriptional output and that substantial functional redundancy exists among FZD receptors in this context (Wang et al., 2016). Importantly, our data indicate that limb shortening and morphogenetic defects occur without overt disruption of β-catenin–dependent signaling, reinforcing the idea that canonical transcriptional readouts alone are insufficient to explain Fzd2-dependent developmental phenotypes. While MEF-based assays demonstrated preserved responsiveness to WNT ligands, these experiments were performed in a fibroblast context and may not fully capture signaling dynamics in limb mesenchyme or chondrocytes. Thus, our *in vitro* findings should be interpreted as evidence of retained signaling competence but they do not exclude the possibility of tissue-specific or spatially restricted differences *in vivo*.

Loss of Fzd2 resulted in reproducible defects in chondrocyte orientation consistent with impaired PCP, yet these phenotypes were not accompanied by substantial changes in VANGL2 phosphorylation. This apparent disconnect highlights an important conceptual point: regulation of PCP is not dictated solely by bulk changes in pathway components. Instead, planar polarity is intrinsically spatial, depending on asymmetric protein localization, directional trafficking, and mechanical integration across tissues (Yang and Mlodzik, 2015). Thus, subtle or localized alterations in PCP signaling can lead to pronounced defects in cellular organization without detectable changes in whole-tissue biochemical readouts. Our findings are consistent with other PCP models, in which morphogenetic defects arise in the absence of dramatic changes regarding VANGL2 phosphorylation or expression.

Our findings in the Fzd2 knockout models are also consistent with emerging models of ADRS pathogenesis, in which disruption of PCP and imbalance between canonical and non-canonical WNT signaling pathways contribute to developmental defects. Functional studies of ADRS-associated variants have demonstrated that mutations in core pathway components, such as DVL1, impair PCP-dependent tissue organization *in vivo* while altering pathway output balance (Gignac et al., 2023). In this context, the increased angular dispersion of chondrocytes observed in the Fzd2 mutants provides a cellular phenotype that aligns with these established mechanisms, even in the absence of overt changes in bulk signaling readouts. Together, these observations suggest that Fzd2 contributes to limb morphogenesis by regulating PCP-dependent cellular organization and further support the idea that disruption of this process represents a common mechanistic feature that underlie ADRS-related phenotypes.

The effects of Fzd2 deletion varied across developmental stages and tissues, with early digit chondrocytes and later femoral chondrocytes distinct sensitivities to Fzd2 loss. Moreover, polarity defects differed between global and limb mesenchyme-specific deletion models, underscoring the context-dependent nature of Fzd2 function. These observations suggest that Fzd2 contributes to limb morphogenesis by fine-tuning cellular organization rather than acting as a uniform regulator of differentiation or proliferation.

Although limb micromass cultures are frequently used to assess chondrogenesis and WNT signaling *in vitro*, we were unable to reliably extend these assays to Cre-mediated deletion of Fzd2 in high-density limb mesenchymal cultures. Adenoviral Cre delivery resulted in substantial cytotoxicity and loss of culture integrity, precluding quantitative assessment of chondrogenic differentiation or signaling outputs. Our analyses of bulk limb tissue demonstrated normal canonical WNT target gene expression and indicate that early chondrogenic differentiation is largely preserved following Fzd2 deletion at early stages. However, we consistently observed reduced *Col10a1* expression at E14.5 across loss-of-function models, suggesting a modest and embryonic stage-specific effect on markers of chondrocyte maturation. Given that these analyses were performed on whole-limb lysates, this reduction could reflect changes in the timing of differentiation, the relative abundance of hypertrophic chondrocytes or gene expression per cell. We, therefore, focused our mechanistic interpretation on *in vivo* phenotypes related to cellular organization and polarity, which provide a more physiologically relevant readout of Fzd2 function during limb development.

The IMPC has reported early embryonic lethality for a distinct Fzd2 allele (Dickinson et al., 2016), which is in contrast to the viability observed following Cre-mediated deletion of the Fzd2 locus in our genetically validated model. This discrepancy may reflect differences in allele design, including the presence of reporter/selection cassettes, background-dependent modifier effects or linked passenger variants that influence viability. Consistent with this, our whole-genome and structural validations support our hypothesis that the *Fzd2^em1Vari^* allele produces an unambiguous *Fzd2* null allele without complex rearrangements, enabling direct attribution of observed phenotypes to Fzd2 loss.

Together, our findings demonstrate that Fzd2 plays a non-redundant role in regulating morphogenetic cell behaviors during limb development while being largely dispensable for bulk canonical Wnt signaling. This distinction has important implications for therapeutic strategies aimed at selectively targeting Wnt receptors, and emphasizes the need for phenotypic assays that capture cellular organization and tissue architecture, rather than relying primarily on transcriptional readouts. More broadly, our work highlights the necessity of rigorous genetic validation, including structural analysis and WGS, when interpreting phenotypes from genetically engineered mice (Diegel et al., 2020). The *Fzd2^em1Vari^* model provides a genetically robust platform for future studies aimed at dissecting receptor-specific functions within the Wnt signaling network.

## MATERIALS AND METHODS

### Experimental animals

The mice used in this study were maintained in accordance with institutional animal care and use guidelines. Experimental protocols were approved by the Institutional Animal Care and Use Committee of the Van Andel Institute. Mice were fed a LabDiet 5021 mouse breeder diet and were housed in Thoren Maxi-Miser IVC caging systems with a 12-h light/12-h dark cycle. Our newly generated B6;C3-*Fzd2^em1Vari^* flox allele is hereafter referred to as *Fzd2^em1Vari^*. The *Fzd2^tm1.1Nat^* mice (Yu et al., 2010, 2012) were obtained from the laboratory of Jeremy Nathans (Johns Hopkins University) and the *Fzd2^tm1Eem^* flox mice (Kadzik et al., 2014) from the laboratory of Edward Morrisey (University of Pennsylvania). The OC-Cre (B6.FVB-Tg(BGLAP-cre)1Clem/J) (Zhang et al., 2002) animals were originally obtained from Thomas Clemens (University of Maryland) and are available from The Jackson Laboratory (JAX stock #019509). The following Cre drivers were purchased from The Jackson Laboratory: B6.C-Tg(CMV-cre)1Cgn/J, JAX stock #006054 (Schwenk et al., 1995); B6.Cg-Tg(Prrx1-cre)1Cjt/J, JAX stock #005584 (Logan et al., 2002); B6.Cg-*E2f1^Tg(Wnt1-cre)2Sor^*/J, JAX stock #022501 (Lewis et al., 2013); B6;CBA-Tg(Sox10-cre)1Wdr/J, JAX stock #025807 (Matsuoka et al., 2005); and B6.FVB-Tg(Pdx1-cre)6Tuv/J, JAX stock #014647 (Hingorani et al., 2003). 129S/Sv-Kras^tm4Tyj^/J, MMHCC strain #01XJ6, JAX stock #008179 (Hingorani et al., 2005) and 129S-*Trp53^tm2Tyj^*/J, MMHCC strain #01XM2, JAX stock # 008652 (Olive et al., 2004) mouse models were originally obtained from the Mouse Models of Human Cancer Consortium (MMHCC; Frederick, MD) repository and are now currently available through The Jackson Laboratory.

### Design of guide RNAs for inserting *loxP* sites

Briefly, guide RNAs (gRNAs) targeting the promoter region of *Fzd2* and the region downstream of the 3′ UTR were designed against the *Mus musculus* (GCA_001632555.1) genome reference sequence (Moreno-Mateos et al., 2015) by using the CRISPOR software version 4.95 (Concordet and Haeussler, 2018) and purchased from Integrated DNA Technologies (Coralville, IA, USA). We used sgRNA 5′-AGGGAGCAGCTTCGCCAGTT-3′ to target the promoter region and sgRNA 5′-GAGCCGGGTTCATTAAAGTT-3′ to target the downstream region of the 3′UTR.

### pNS20-SpCas9-mSA construct and recombinant protein generation and purification

To generate pNS20-SpCas9-mSA, we PCR amplified the optimized linker and mSA-coding sequence from the pCS+Cas9-mSA plasmid, created by the laboratory of Janet Rossant (Addgene #103882) (Gu et al., 2018). A BamHI site was incorporated to the 5′ end of the mSA fragment and a MluI site to the 3′ end. We modified the original BamHI site present at the 3′ end of the mSA fragment from a GGA to a GGT codon to eliminate the restriction site but retain the glycine. BamHI and MluI restriction sites were used to remove the SNAP coding sequence and insert the mSA coding sequence into pNS20-SpCas9-SNAP (Laboratory of Gerald Schwank, Addgene #113717) (Savic et al., 2018). Recombinant protein was generated and purified as previously described (Savić et al., 2019). Briefly, N-terminally His6-MBP-tagged Cas9 mSA was expressed in *E. coli* Rosetta (DE3) pLysS cells (Novagen) in 6L of LB medium to an OD_600_ of ∼1 at 30°C and induced with 100 μM isopropyl-β-D-thio-galactopyranoside (IPTG) at 16°C overnight. For purification of the Cas9 fusion protein, cells were harvested, resuspended and lysed in 20 mM Tris pH 8.0, 500 mM NaCl, 5 mM imidazole, using a French Press with pressure set to 900 Pa. Lysates were cleared by centrifugation for 1 h at 38,700 ***g*** and passed over a 5 ml HisTrap HP column (Cytiva, formerly GE Healthcare). The column was washed with 15 column volumes of 20 mM Tris pH 8.0, 500 mM NaCl and 5 mM imidazole, and then eluted with 20 mM Tris pH 8.0, 250 mM NaCl, 250 mM imidazole. To the eluted protein, 1 mg of TEV protease was added for each 25 mg of Cas9 and dialyzed together against 20 mM HEPES pH 7.5, 100 mM KCl at 4°C overnight. The next morning the cleaved protein was loaded on a 5 ml HiTrap HeparinHP column (GE Healthcare). The protein was eluted over a 100 mM to 2 M KCl gradient. The protein was further purified by size-exclusion chromatography through a HiLoad 26/60 Superdex 200 column (GE Healthcare) in 20 mM HEPES pH 7.5, 250 mM KCl. DTT was added to 2 mM and glycerol to 10% to the final purified protein for storage.

### *In vitro* DNA cleavage by recombinant Cas9-mSA protein

The DNA cleavage activity of our Cas9-mSA endonuclease was assayed on linearized plasmid DNA containing eGFP. The plasmid was digested overnight with XbaI followed by column purification (28104, Qiagen). We used RNA guide 5′-GTGAACCGCATCGAGCTGAA-3′ to target eGFP (Mali et al., 2013), which was in complex with our Cas9-mSA enzyme *in vitro*. The cleavage assay was performed as previously described (Mehravar et al., 2019). In short, the cleavage reaction contained the following components: 1 μl sgRNA (1 μg μl^−1^), 3 μl 10× Cas9 nuclease reaction buffer (1 M NaCl, 0.1 M MgCl_2_, 0.5 M Tris-HCl, 1 mg ml^−1^ BSA pH 7.9), 600 ng of either wild-type Alt-R™ S.p Cas9 nuclease (cat. #1081058; Integrated DNA Technologies) or S.p Cas9-mSA protein, 500 ng of linearized plasmid, and double-distilled H_2_O to a final volume of 30 μl. The mixtures were incubated at 37°C for 2 h, after which 1 μl proteinase K (20 mg ml^−1^) was added, and the mixture was subsequently incubated at 65°C for 10 min to release the DNA from the Cas9 protein. Mixtures lacking the sgRNA targeting eGFP were used as a negative control.

### BIO-PCR donor template

Double-stranded DNA fragments with ∼1 kb spanning homology arms around a *loxP* sequence were synthesized as gBlocks Gene Fragments (Integrated DNA Technologies). The donor template had a disrupted PAM sequence and a unique *Xho*I restriction site in the 5′ donor template along with an *Age*I restriction site in the 3′ donor template. 5′ biotin-modified oligonucleotides were used to PCR amplify the biotinylated template, which then underwent PCR cleanup and ethanol precipitation before microinjection (Gu et al., 2018).

### Microinjection and embryo transfer

Embryos were generated by intercrossing B6C3F1/J animals (The Jackson Laboratory, JAX stock #100010). Thirty 3−4-week-old females were superovulated by intraperitoneal (IP) injection of pregnant mare serum gonadotropin (5 IU) followed by IP injection of human chorionic gonadotropin (5 IU) 48 h later. Mating was established on the same day. The B6C3F2 two-cell embryos used for injection were collected at two different time points, with 50% recovered at 0.5 days post coitum (dpc) and the other 50% at 1.5 dpc. Embryos recovered at 0.5 dpc were cultured for 24 h in Research Vitro Cleave Medium (K-RVCL) (Cook Medical, cat #G38849), while those collected at 1.5 dpc were placed in K-RVCL and microinjected within 6 h after recovery.

Microinjection commenced immediately following recovery of the 1.5 dpc embryos. A modified mouse zygote microinjection technique using the Alt-R™ CRISPR-Cas9 System ribonucleoprotein delivery protocol (Integrated DNA Technologies) was used. Complete sgRNAs from Integrated DNA Technologies (crRNA and tracrRNA) were incubated with Cas9-mSA protein to generate ribonucleoprotein (RNP) complexes before the biotinylated template (BIO-template) was added (Harms et al., 2014). Both complexes were combined for a final microinjection mix containing 12.5 ng μl^−1^ sgRNA: Cas9-mSA protein, 4 ng μl^−1^ BIO-template targeting both the 5′ and 3′ regions in injection buffer (1 mM Tris-HCl pH 7.5; 0.1 mM EDTA). RNP complexes were injected into each nucleus of 2-cell embryos under negative capacitance generated via MICRO-ePORE pinpoint cell penetrator (World Precision Instruments). Embryos were microinjected in paraffin oil-covered M2 medium (cat #M2113, CytoSpring, Mountain View, CA, USA) and then transferred to K-RVCL for culture. Twenty-four hours later, 12−15 of these ∼8-cell embryos were transferred unilaterally into pseudopregnant females. Founders were identified by standard PCR methods and backcrossed once to wild-type C57BL/6J mice before intercrossing to generate the animals used in our study.

### Genotyping by PCR and insertion−deletion sequencing

Genomic DNA from mouse tail biopsies was isolated by alkaline digestion. To genotype the *Fzd2^em1Vari^* flox 5′ *loxP* allele we used primers Fzd2-5′loxP-Fwd (5′-GAGATTACAGGTGTGAGCTACTG-3′) and Fzd2-5′loxP-Rev (5′-CTGGAGAGGGAAGGGAATTTG-3′) to amplify a 367-bp wild type product and a 398-bp *loxP* product. To genotype the 3′loxP allele we used primers Fzd2-3′loxP-Fwd (5′-GAGAAAGCAGGAGGATGTAAGAG-3′) and Fzd2-3′loxP-Rev (5′-CTGTCCCACCTTCCATCAAAT-3′) to amplify a 292-bp wild-type product and a 324-bp *loxP* product. To determine Cre-mediated recombination both *in vitro* and *in vivo*, we used the following primers: Fzd2-5′loxP-Fwd, Fzd2-3′loxP-Fwd and Fzd2-3′loxP-Rev to amplify a 485-bp knockout product, a 324-bp *loxP* product and a 292-bp wild-type product. For each model all PCR products were Sanger sequenced (GENEWIZ from Azenta Life Sciences, South Plainfield, NJ, USA) for validation and to confirm correct *loxP* insertion. SnapGene software (www.snapgene.com) was used for sequence alignment and animal model design.

To genotype the *Fzd2^tm1Eem^* flox allele, we used the following primers as previously described by Kadzik et al. (2014): Fzd2-Flox_F (5′-GCCTGCTCGCTATTTTTGTTGGC-3′) and Fzd2-Flox_R (5′-AAATGAGGAGGGAGAAAGAGGGGG-3′) to amplify a 210-bp wild-type and 300-bp *loxP* product.

### Generation of *Fzd2^em1Vari^* knockout alleles

To generate global knockout animals, male mice homozygous for the *Fzd2^em1Vari^* floxed allele were crossed with female B6.C-Tg(CMV-cre)1Cgn/J (The Jackson Laboratory, JAX stock #006054) (Schwenk et al., 1995). The resulting offspring were genotyped to determine allele-specific Cre-mediated recombination for Fzd2, using primers Fzd2-5′loxP-Fwd (5′-GAGATTACAGGTGTGAGCTACTG-3′) and Fzd2-3′loxP-Rev (5′-CTGTCCCACCTTCCATCAAAT-3′) to amplify a 485-bp knockout product. PCR products were Sanger sequenced by GENEWIZ (Leipzig, Germany) to validate correct PCR products, insertion−deletion (indel) insertion and recombination.

In a similar manner, we generated global mutants for the *Fzd2^tm1Eem^* allele. We designed a primer upstream of the 5′ loxP site within the 5′ UTR to determine Cre-mediated recombination within this allele. We used the following primers for determining Cre recombination: Fzd2-AS-F (5′-AAACCGACTAATTGGGATCGG-3′) along with Fzd2-Flox_F (5′-GCCTGCTCGCTATTTTTGTTGGC-3′) and Fzd2-Flox_R (5′-AAATGAGGAGGGAGAAAGAGGGGG-3′) R to amplify a 474-bp knockout, 210-bp wild-type and 300-bp *loxP* product.

### Restriction digestion of PCR products

PCR products for the 5′ *loxP*, 3′ *loxP* and Cre-mediated KO recombination allele were amplified from the *Fzd2^em1Vari^* flox model, column purified (New England Biolabs, #T1030S) and then used for a restriction endonuclease digest. Digestion reactions consisted of 500 ng of PCR product and 20 units of either *Xho*I (R1046S, NEB) or *Age*I (R3552S, NEB) in their specified buffers. The reactions were incubated at 37°C for 1 h before samples were separated by electrophoresis on a 2% agarose gel.

### Whole-genome sequencing and ONT long-read sequencing

Genomic DNA (gDNA) was extracted from whole blood via cardiac puncture from 6−8-week-old *Fzd2^tm1Eem^* (homozygous flox or homozygous global mutants created by crossing with CMV-cre mice) or *Fzd2^em1Vari^* (homozygous flox) animals using Quick-DNA™ Miniprep Plus Kit (Zymo Research). Illumina libraries were constructed with average insert sizes of ∼800 bp and sequenced on an Illumina NovaSeq6000 using paired-end 150 bp reads, and an average of 30× coverage was achieved (Illumina, San Diego, CA, USA). The resulting reads were aligned with a BWA-MEM aligner (Li and Durbin, 2009) to mm10 mouse reference genome (UCSC Genome Browser) for sequence and structural accuracy. Nanopore long-read sequencing was performed using the same *Fzd2^tm1Eem^* homozygous flox/flox genomic DNA used for Illumina sequencing on a MinION flow cell (Oxford Nanopore Technologies, Oxford, UK). The resulting reads were aligned to mm10 mouse reference genome (UCSC Genome Browser) by using the Minimap2 aligner (Li, 2018). The resulting binary alignment map (BAM) files were visualized with Integrative Genomics Viewer (The Broad Institute, Cambridge, MA, USA).

### Generation and validation of the *Fzd2^KO-delta-Cterm^* allele

The improved genome editing of oviductal nucleic acid delivery (i-GONAD) technique was used to edit the existing *Fzd2^tm1.1Nat^* allele (Ohtsuka et al., 2018; Gurumurthy et al., 2019). The two sgRNAs were designed to target the *Fzd2^tm1.1Nat^* remaining targeting vector (sgRNA1, 5′-CGGGCATGTTGGCGCGATAT-3′) (Integrated DNA Technologies) and the Fzd3 3′UTR (sgRNA2, 5′-GGCACGGTGCAGTCCGGACC-3′, (both from Integrated DNA Technologies). The night before surgery, B6C3F1/J females (The Jackson Laboratory, JAX stock #100010) were mated to *Fzd2^tm1.1Nat^* homozygous knockout (KO/KO) males. Vaginal plug detection was performed at 9 AM the following morning. RNP complexes were made using Alt-R^®^ S.p. Cas9 Nuclease V3 (1 μg/μl^−1^), sgRNA1 (30 μM), and sgRNA2 (30 μM). Surgery was performed at approximately E0.7 (17:00). Complete details regarding the i-GONAD protocol are available (Gurumurthy et al., 2019). Briefly, females were anesthetized using Avertin (240 mg kg ^-1^; intraperitoneal) (2,2,2-tribromoethanol, Sigma-Aldrich, cat #T48402; 2-Methyl-2-butanol, Sigma-Aldrich, cat #240486) and Rimadyl® (Carprofen; 5 mg kg^−1^; subcutaneous) (Zoetis, cat #10022998) was administered for analgesia. Dorsally, an incision (10 mm) was made at the central portion of the back, followed by two small (5 mm) internal incisions on the right and left to expose the ovaries. The right ovary was pulled from the body cavity and 1.5 μl of the RNP complex was administered to the oviduct. A sterile Kimwipe soaked in 1× PBS was draped over the injected oviduct and the Kimwipe-covered oviduct was electroporated using electrokinetic tweezers (#CUY652P2.5x4; NepaGene, Ichikawa, Japan) with eight pulses at 50 V, 5 ms per pulse and 1 s interval in between pulses (BTX T820 square wave electroporator, cat #80264-1, Artisan Technology Group, Champaign, IL, USA). After the ovary was placed back into the body cavity, the procedure was repeated on the left side. The external incision was closed with sterile surgical staples and the animals recovered on a heating pad. Post-operative carprofen was administered as needed.

Offspring from two females were genotyped using common forward (5′-GGCATTGACCCTGAGTGATT-3′) and *Fzd2^tm1.1Nat^* reverse (5′-GGAAGGATGTACCGATGAACAG-3′) or *Fzd2^KO-delta-Cterm^* reverse (5′-GGAACTAACTCCCAACTCCTTT-3′) primers. Two founder animals were then crossed with wild-type C57BL/6J to confirm germ-line transmission. Heterozygous mice were intercrossed using timed matings to collect pups at E18.5 for limb length analyses.

### Limb micromass cultures

Limbs were harvested from E11.5–E12.5 *Fzd2^tm1Eem^* or *Fzd2^em1Vari^* embryos for micromass culture according to previously published protocols (Iezaki et al., 2019; Underhill et al., 2014) with modifications. Briefly, fore- and hindlimbs were removed from embryos and digested in ∼10 U ml^−^ of Dispase II (Gibco) in DMEM (Gibco) at 37°C for 1 h. Yolk sacs were collected for genotyping. Limbs were further dissociated by pipetting up and down and then centrifuged at 300 ***g*** for 5 min. Cell pellets were resuspended in culture medium (DMEM, 4.5 g l^−1^ glucose, 1 mM sodium pyruvate, 2 mM L-glutamine, 10% FBS, 1% penicillin–streptomycin) and passed through a 40-μm cell strainer. Cells were centrifuged at 300 ***g*** for 5 min and resuspended in culture medium to reach a cell concentration of 10−20×10^6^ cells ml^−1^. Cells were plated in a 10 μl micromass drop in a 24-well tissue culture well and incubated at 5% CO_2_, 37°C tissue culture incubator for 2 h to allow cell attachment followed by the addition of 1 ml of culture medium. The following day, the cells were gently washed with 1× PBS and incubated for 1 h with Ad5CMV:eGFP or Ad5CMV:Cre-eGFP [multiplicity of infection (MOI) =100, University of Iowa Viral Vector Core Facility]. Culture medium was added to inactivate viral complexes. For confirmation of viral infection, cells were imaged for eGFP incorporation 48 h later and gDNA was collected for Sanger Sequencing by GENEWIZ or cell suspensions for flow cytometric analyses. Alternatively, RNA was extracted from micromass cultures for qPCR 72 h after Ad-Cre transduction.

### Quantitative real-time polymerase chain reaction

RNA was extracted from *Fzd2^tm1Eem^* micromass cultures using Quick-RNA Miniprep kit (Zymo Research) followed by cDNA conversion using High-Capacity cDNA Reverse Transcription Kit (Applied Biosystems). Real-time quantitative PCR (RT-qPCR) was performed with SYBR green master mix (Life Technologies) on StepOnePlus™ Real-Time PCR System (Applied Biosystems). Two sets of Fzd2 primers were used to target either the Fzd2 CDS region or 3′ untranslated region (UTR) upstream of the *loxP* insertion site in both Fzd2-flox models, i.e. mFzd2-CDS-F (5′-GCTGCGCTTCCACTTTCTTC-3′) and mFzd2-CDS-R (5′-GAACGAAGCCCGCAATGTAG-3′) or mFzd2-3UTR-F (5′-GCACCCTACGGACTCCTATT-3′) and mFzd2-3UTR-R (5′-GGTGTTGGGCAGAGTTCTTT-3′), respectively.

For limb expression studies, limbs were collected from *Fzd2^tm1.1Nat^*, *CMV-Cre-Fzd2^em1Vari^*, and *Prx1-Cre-Fzd2^em1Vari^* animals at E12.5−E14.5. RNA was extracted from limb tissue using Quick-RNA Miniprep kit (Zymo Research) followed by cDNA conversion using High-Capacity cDNA Reverse Transcription Kit (Applied Biosystems). Real-time quantitative PCR was performed with SYBR green master mix (Life Technologies) on QuantStudio™ 6 Pro Real-Time PCR System (Applied Biosystems). Primers from Integrated DNA Technologies included: mRibo-F 5′-TTAAGCGAAACTGGCGGAAAC-3′; mRibo-R 5′-TTGTTGCTCCCATAACCGATG-3′; mAxin2-F 5′-TCATTTTCCGAGAACCCACCGC-3′; mAxin2-R 5′-GCTCCAGTTTCAGTTTCTCCAGCC-3′; mSox9-F 5′-AGAACAAGCCACACGTCAAG-3′; mSox9-R 5′-CAGCAGCCTCCAGAGCTT-3′; mCol2a1-F 5′-GTGGACGCTCAGGAGAAACA-3′; mCol2a1-R 5′-TGACATGTCGATGCCAGGAC-3′; mCol10a1-F 5′-TTCTGCTGCTAATGTTCTTGACC-3′; mCol10a1-R 5′-GGGATGAAGTATTGTGTCTTGGG-3′; mAcan-F 5′-GTGGAGCCGTGTTTCCAAG-3′; and mAcan-R 5′-AGATGCTGTTGACTCGAACCT-3′. The following primers were designed and ordered from GeneCopoeia: mFzd1-F 5′-GCGCTCATGAACAAGTTCGG-3′; mFzd1-R 5′-ACTCTGGTAGCAAGGAGGGA-3′; mFzd2-F 5′-GCTGCGCTTCCACTTTCTTC-3′; mFzd2-R 5′-GAACGAAGCCCGCAATGTAG-3′; mFzd3-F 5′-TTCACTATGGCTGGCAGTGT-3′; mFzd3-R 5′-AGTTAGAGTTCCGGGGATGC-3′; mFzd4-F 5′-TGAACTGACTGGCTTGTGCT-3′; mFzd4-R 5′-CGCAATGAACAGCGTTCCAA-3′; mFzd5-F 5′-TGCTTCATCTCCACGTCCAC-3′; mFzd5-R 5′-TGACACACACAGGTAGCACG-3′; mFzd6-F 5′-CGGGAGAAAATGGAGTTGCG-3′; mFzd6-R 5′-GAGGCGTCCAGGTCATACAG-3′; mFzd7-F 5′-GCGAGAAAGGCATCTCGGTA-3′; mFzd7-R 5′-CCAGAGGGTAGAACTGGTGC-3′; mFzd8-F 5′-ACCAGAGCCTTGACAACCTACG-3′; mFzd8-R 5′-GCTGCTTGATGACTGAACGGA-3′; mFzd10-F 5′-GCTCACCTTCCTCATCGACC-3′; and mFzd10-R 5′-TAGCCCACCGAATAAACGCA-3′.

### Flow cytometry

Single-cell suspensions were generated from limb micromass cultures or from limb buds dissected from E12.5 embryos for flow cytometric analyses. Limb micromass cultures were trypsinized in TrypLE (Gibco) and neutralized with medium containing FBS. Following centrifugation at 300 ***g*** for 5 min, cells were resuspended in cold flow buffer (1× HBSS without magnesium, calcium or Phenol Red, with 2% FBS), and cells were filtered through a pre-wet 40um cell strainer into a 50 ml conical tube.

Single-cell suspensions from limb buds were prepared following a modified protocol (Nusspaumer et al., 2017; Reinhardt et al., 2019). Briefly, limb buds from E12.5 mouse embryos were dissected and digested in 1 ml 1× HBSS (without magnesium, calcium, or phenol red) containing 1 mg ml^−1^ collagenase D (Millipore Sigma) and 50 μg ml^−1^ DNase I (Roche) at 37°C for ∼45 min. Limb buds were gently pipetted every 5 min with a 1000-μl pipette tip until the tissue was dissociated into a single-cell suspension. Cold flow buffer was added to stop the digestion, and the cell suspension was filtered through a pre-wet 40 μm cell strainer into a 50 ml conical tube and centrifuged at 300 ***g*** for 10 min.

Approximately 2×10^5^ (micromass) or 1×10^6^ (limb bud digestion) cells were stained with 50 nM non-conjugated anti-FZD2 antibody [for methods for antibody generation see Tao et al. (2019)] for 30 min on ice. Cells were washed and stained with Alexa Fluor® 647 AffiniPure® F(ab′)₂ Fragment Goat Anti-Human IgG (1:1000, cat #109-546-097, Jackson ImmunoResearch) for 30 min on ice. Cells were washed and resuspended in 200 μl flow buffer containing 5 μg ml^−1^ DAPI prior to analyses. Alternatively, if cells were fixed prior to analysis, they were stained using the Zombie Violet™ Fixable Viability Kit (1:1000, cat #100002, BioLegend) for 30 min on ice during secondary antibody staining. Cells were then washed and fixed in 4% paraformaldehyde (PFA) on ice for 20 min, washed and resuspended in 200 μl of flow buffer. Cells were analyzed on CytoFLEX S (Beckman Coulter).

### Copy number detection

For copy number detection, gDNA was harvested from limb buds from E14.5 *Fzd2^tm1.1Nat^* or lung tissue from *Fzd2^tm1Eem^* 8-week-old mice using Quick-DNA™ Miniprep Plus Kit (Zymo Research). A modified PCR protocol was used (D'Haene et al., 2010). Briefly, the genomic DNA was diluted to ∼30 ng μl^−1^, and 2 μl was used in a 20 μl PCR reaction. Fzd2 Cq values were normalized to Fzd1, which was considered as an internal reference. The primers used are the same as those for limb bud qPCR described above.

### *Fzd2^tm1.1Nat^* C-terminal peptide studies

The truncated mouse Fzd2 gene (Fzd2-delN) was cloned from pRK5-mFzd2 (Addgene #42254) with a 1D4 tag at the C-terminus by PCR using primers SalI-mFzd2-5del-F (5′-AATAGTCGACGCCATGGGCCAGATCGAC-3′) and NotI-1D4-R (5′-AAGCAGCGGCCGCTTAGGCAGGCGCCACTTG-3′). The resulting plasmids were transfected into HEK293T or HEK293T-FZDless (Eubelen et al., 2018) (gift from Dr Benoit Vanhollebeke, Université libre de Bruxelles, Belgium) expressing a Wnt reporter gene (super top flash, STF) with X-tremeGENE HP DNA Transfection Reagent (Roche). The luciferase activity was detected 48 h after transfection using the luciferase assay system (Promega) and a BioTek Synergy Neo Microplate Reader (BioTek).

### Micro-computed tomography

Limbs and skulls from E18.5 animals were fixed in 10% neutral buffered formalin (NBF) at room temperature for 48 h, followed by storage in 70% ethanol. Skulls and Fore- and hindlimbs were scanned at an X-ray voltage of 50 kV, a current of 201 μA and with a 0.5 mm aluminum filter using the SkyScan 1172 micro-computed tomography (MicroCT) system (Bruker MicroCT: Kontich, Belgium). A pixel resolution of 2000×1200 and voxel size of 8 μm were used. Images were reconstructed using NRecon program version 1.7.4.6 (Bruker). A volume of interest (VOI) (DataViewer software version 1.5.6.3) and region of interest (CTAn software version 1.18.8.0) were defined for each sample. A 3D-rendered model was visualized in CTVol software version 2.2.1.0. We used distinct proximal and distal anatomical landmarks to determine bone lengths in CTan for the following bones: femur, tibia, fibula, humerus and ulna. The built-in measurement tool in CTAn was utilized to calculate the distance between image slices, which represents the bone length. Skull measurements included skull length, pre-maxilla length, pre-maxilla width, mandible length, mandible width and nasal bone length. These measurements were performed using 3D Slicer (Fedorov et al., 2012).

Femurs from 3-month-old male and female mice were fixed in 10% NBF at room temperature for 48 h, stored in 70% ethanol and scanned as indicated above. The adult femur sample settings included: 60 kV, 167μA, 0.5 mm aluminum filter, 2000×1200 pixel resolution and 7-μm image voxel size. Femoral images were reconstructed, and VOI/ROI defined as indicated above. For trabecular analyses, the ROI was defined as a 2.5 mm long region in the distal epiphysis starting 0.25 mm proximal to the growth plate. The cortical ROI was defined as 0.6 mm in height as the central metaphysis. The ROIs were analyzed using the CTAn 1.18.8.0 program. Trabecular parameters measured included: bone mineral density (BMD), bone volume/tissue volume (BV/TV), trabecular thickness (Tb.Th), trabecular separation (Tb.Sp) and trabecular number (Tb.N). Cortical parameters included: tissue mineral density (TMD), tissue area (T. Ar), bone area (B.Ar), cortical area fraction (CAF) and cross-sectional thickness (Cs. Th).

### Wheat germ agglutinin staining, cell elongation and orientation analysis

E12.5 forelimbs were collected, fixed overnight in 4% PFA (Electron Microscopy Sciences, Hatfield, PA, USA) at 4°C and embedded in Tissue-Tek® O.C.T. Compound (cat #4583, Sakura Finetek, Torrance, CA, USA). Samples were stored at −80°C prior to cryosectioning. Samples were sectioned (5 μm), washed three times with 1× PBS, and stained with 1 mg ml^−1^ wheat germ agglutinin (WGA) fluorescent CF594 dye conjugate (cat #29023-1, Biotium, Fremont, CA, USA) for 10 min at room temperature. Slides were washed three times with 1× PBS then coverslips were mounted with ProLong Gold antifade reagent with DAPI (Invitrogen). Tissue samples were imaged using the Zeiss AxioScan7 Fluorescent Slide Scanner. Individual cell length-to-width ratios and cell orientation were measured for the chondrocytes within the middle of the digit.

E18.5 femurs were fixed in 10% formalin and embedded paraffin using standard protocols. Samples were sectioned and stained for hematoxylin and eosin (H&E), followed by imaging using the Zeiss AxioScan7 Fluorescent Slide Scanner. Individual cell orientations were measured for the chondrocytes within growth plate of the femur.

### Isolation and differentiation of osteoblastic cells from mouse calvaria

Calvariae were isolated from 4-day-old *Fzd2^em1Vari^* homozygous flox (fl/fl) pups following standard protocols (Zhong et al., 2016). Briefly, following isolation of calvariae under sterile conditions, calvariae were rinsed in 1× PBS at 37°C for 30 min, while shaking on a orbital shaker at 90 rpm. The PBS was removed and the calvariae were rinsed two additional times under the same conditions. After the third wash, the PBS was removed, and 10 ml of Type 1 Collagenase (200 U ml^−1^ in 1× PBS, filter sterilized, Worthington Biochemical Corp., Cat. no. 4197) was added. The calvaria were digested in the collagenase solution while shaking at 90 rpm for 10 min at 37°C. The collagenase solution was removed and digestion was repeated once. The calvariae were then digested with fresh collagenase solution while shaking at 90 rpm for 15 min at 37°C. This solution was removed from each calvaria, strained though a 70-μm cell strainer (BD, cat. no. 352350), and collected. This digestion and collection step was repeated twice and the collected cells were combined. The pooled digestions were centrifuged for 5 min at 300 ***g***. The pellet was resuspended in Minimum essential medium, alpha 1x (αMEM; Corning, cat. no. 10-022-CV) with 10% FBS/100 U ml^−1^ penicillin−streptomycin and plated at a density of 2500−5000 cells cm^−2^. Cells were cultured at 37°C until reaching ∼75% confluency. Cells were infected with adenovirus containing Cre (Ad5CMV:Cre-eGFP; MOI =100) or control adenovirus (Ad5CMV:eGFP; MOI =100). Cells were split for osteoblast differentiation experiments 48 h later, and cultured in mineralization medium (αMEM supplemented with 10% FBS, 100 U ml^−1^ penicillin−streptomycin, 50 μg ml^−1^ ascorbic acid and 10 mM β-glycerophosphate). Cells were stained with Crystal Violet or alkaline phosphatase (NBT/BCIP Substrate Solution, Thermo Fisher Scientific) on days 7, 14 and 21. Plates were scanned and ALP was quantified using ImageJ.

### Western blot analysis

Mouse embryonic fibroblasts (MEFs) were generated from *CMV-Cre–Fzd2^em1Vari^* embryos and maintained in Dulbecco's modified Eagle medium (DMEM; Gibco) supplemented with 10% fetal bovine serum (FBS; Gibco) and 1% penicillin–streptomycin at 37°C with 5% CO_2_. For Wnt stimulation experiments, cells were plated at equal density, treated with 1 μM LGK-974 for 48 h, then treated for 24 h with control conditioned medium (CTRL CM; diluted 1:3 in MEF culture medium), WNT3A-conditioned medium (diluted 1:3 in MEF culture medium), or WNT5A-conditioned medium (diluted 1:3 in MEF culture medium). CM was made in DMEM supplemented with 10% fetal bovine serum and 1% penicillin-streptomycin according to manufacturer's instructions (ATCC). CTRL CM was generated using parental L cells (ATCC, CRL-2648); WNT3A CM was generated using L-Wnt-3A cells (ATCC, CRL-2647); and WNT5A CM was generated using L-Wnt-5A cells (ATCC, CRL-2814).

For *in vivo* analyses, forelimbs and hindlimbs were dissected from *Fzd2^tm1.1Nat^* and *CMV-Cre–Fzd2^em1Vari^* embryos at E12.5. Limb tissues were flash frozen prior to protein extraction. Cells and tissues were lysed in buffer containing 50 mM Na_2_HPO_4_, 1 mM sodium pyrophosphate, 20 mM NaF, 2 mM EDTA, 2 mM EGTA, 10 mM NaCl, 1% Triton X-100, 1 mM DTT and supplemented with protease and phosphatase inhibitor cocktails (Roche). Lysates were cleared by centrifugation at 14,000 ***g*** for 15 min at 4°C, and protein concentration was determined using a BCA assay (Thermo Fisher Scientific). Equal amounts of total protein were separated by SDS–PAGE and transferred to PVDF membranes.

Membranes were blocked in EveryBlot Blocking Buffer (Bio-Rad) and incubated overnight at 4°C with primary antibodies recognizing activated β-catenin (ABC, Cell Signaling Technologies, #8814), phosphorylated LRP6 (Cell Signaling Technologies, #2568), VANGL2 phosphorylated at residues T79/S79/S82 (Sigma, #SAB5701945) or residues S79/S82/S84 (Invitrogen, #MA5-38242), ROR1 (Cell Signaling Technologies, #16540), ROR2 (Cell Signaling Technologies, #88639), DVL3 (Cell Signaling Technologies, #3218) and cleaved caspase-3 (Cell Signaling Technologies, #9664). β-ACTIN (Cell Signaling Technologies, #5125) and GAPDH (Cell Signaling Technologies, #3683) were used as loading controls. For densitometric analysis, band intensities were quantified using Image Lab software (Bio-Rad). Target protein signals were normalized to those of the corresponding loading control and values were expressed relative to wild-type samples within each experiment.

### Statistical analyses

Limb length and skull measurement data were analyzed using unpaired two-tailed Student's *t*-test to compare knockout or conditional knockout samples to wild-type samples. Ordinary one-way ANOVA with Dunnett's post hoc multiple comparisons test was performed using microCT data of adult 3-month-old animals to compare heterozygotes and homozygotes to wild-type samples. Linear mixed-effect models assessed differences in normalized qPCR values across genotypes. A random effect for animal ID was included, and models were stratified by Fzd. All results were adjusted for multiple testing using the Benjamini−Hochberg method. Linear mixed-effects regressions were used to model length to width ratios and cell orientation by genotypes. We used a random effect for animal ID and stratified the models by line. *P*-values were adjusted for multiple testing using the Benjamini−Hochberg method. The Kolmogorov–Smirnov test was used on the *n*=1 vs *n*=1 dataset to assess distributional differences between genotypes. A *P*-value of <0.05 was considered significant. The data presented are the mean±standard deviation (s.d.). No samples were excluded from analyses.

### Use of AI tools

We used ChatGPT for assistance in writing and editing the manuscript.

## Supplementary Material

10.1242/dmm.052410_sup1 Supplementary information
