# Supplementary material for: Clarifying Frizzled 2 function in development through genetically validated mouse models
Source: Dis Model Mech. 2026 Jun 26;19(7):dmm052410. doi: 10.1242/dmm.052410 (PMC13446565; doi:10.1242/dmm.052410)
Supplement: Supplementary file 1 [file dmm-19-052410-s1.pdf]

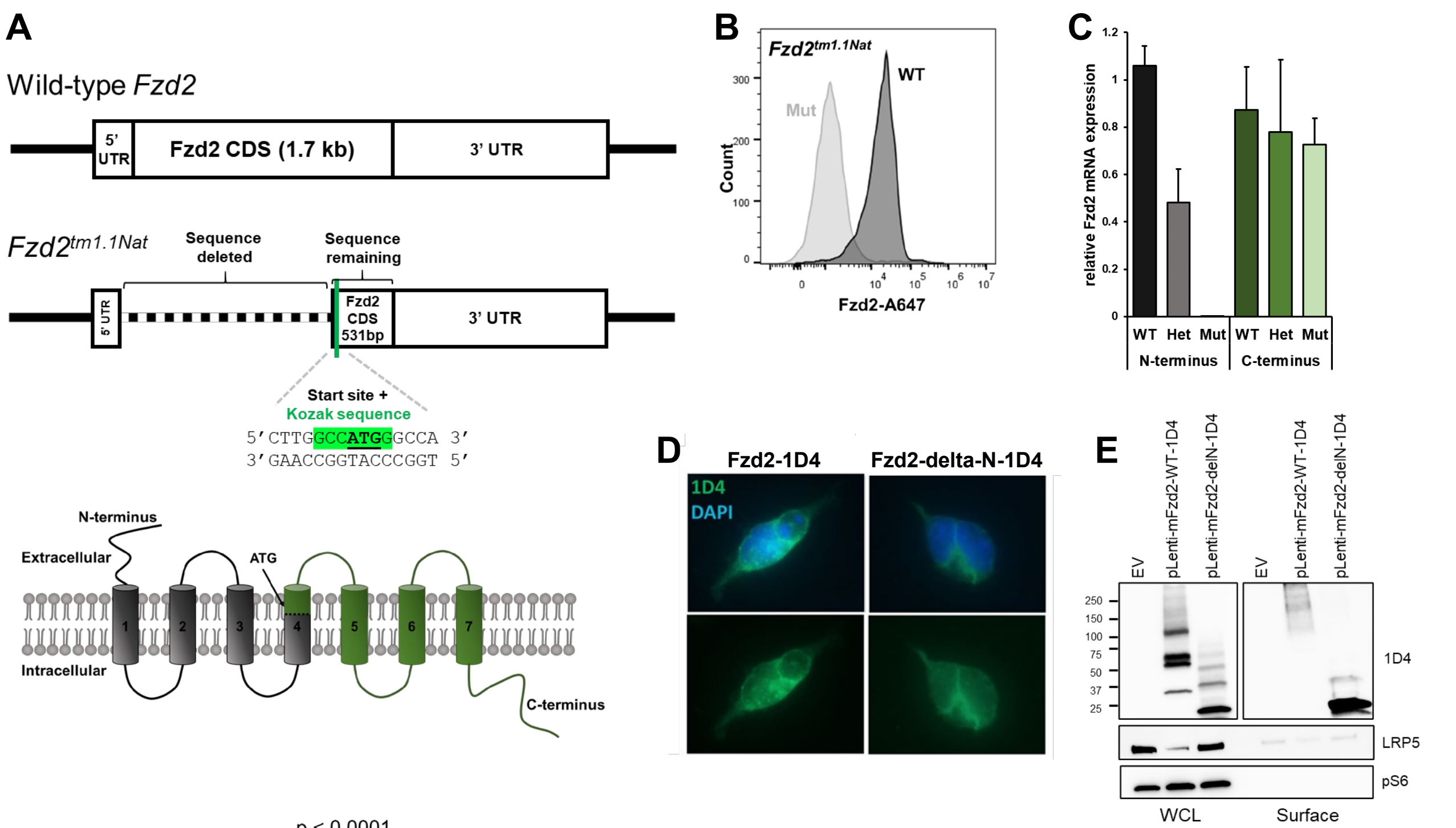


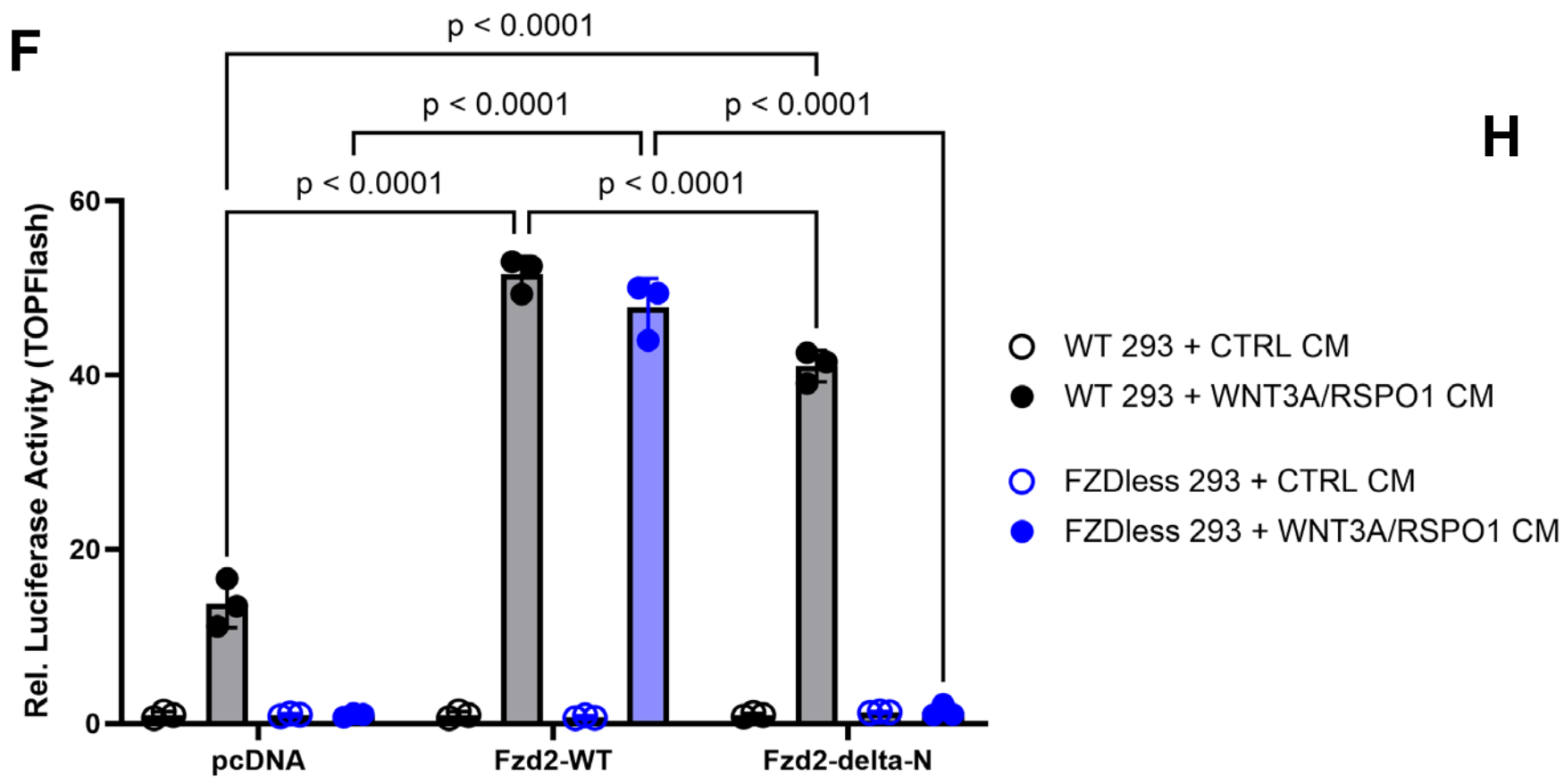


## H *Fzd2-KO-delta-Cterm*

| | WT | HET | KO |
|---|---|---|---|
| Embryos | 7 | 8 | 17 |
| % of total | 22% | 25% | 53% |
| Cleft palate | 0 | 0 | 4 |

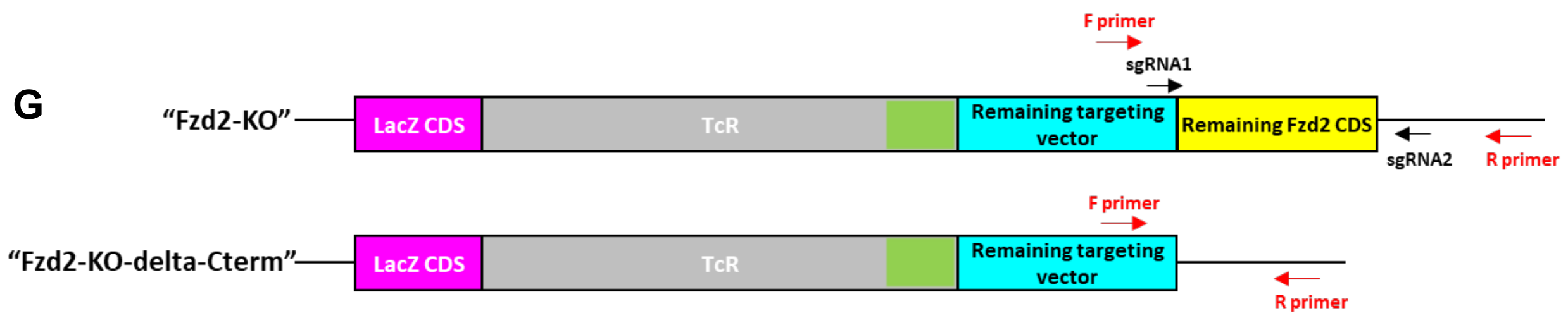


Disease Models & Mechanisms • Supplementary information

**Fig. S1. *Fzd2$^{tm1.1Nat}$* allele retains functional C-terminal peptide.** (**A**) *Fzd2$^{tm1.1Nat}$* allele retains 531 bp of Fzd2 C-terminal sequence that could encode a protein with an in-frame ATG and Kozak sequence (green). (**B**) Flow cytometry of E14.5 limb bud cells using antibody targeted to N-terminal FZD2. Cells were gated to discard debris and doublets, and live cells were determined by DAPI exclusion. FZD2 median fluorescent intensity (MFI) was compared between cells wild-type (WT) or homozygous (Mut) for the *Fzd2$^{tm1.1Nat}$* allele. WT MFI = 18294; Mut MFI = 1267 (**C**) qRT-PCR of E14.5 limb buds from littermates wild-type (WT), heterozygous (Het), or homozygous (Mut) for the *Fzd2$^{tm1.1Nat}$* allele. The relative transcription of either the N-terminal (grey bars) or C-terminal (green bars) portions are compared. Note the C-terminal PCR primers are located in the remaining Fzd2 sequence. (**D**) Immunofluorescence for the 1D4 epitope reveals similar localization of full-length Fzd2 (Fzd2-1D4) and the C terminal fragment (Fzd2-delta-N-1D4). (**E**) A plasmid directing expression of the C-terminal fragment (Fzd2-delta-N) produces a stable protein when ectopically expressed in HEK293T cells (detected with a 1D4 tag). Surface biotinylation shows expression of the truncated Fzd2-delta-N at the surface. (**F**) Wild-type (WT) HEK293 cells or HEK293T cells lacking all 10 FZDs (FZD-less) were transfected with pcDNA-based plasmids expressing empty vector (pCDNA), Fzd2-WT or Fzd2-delta-N. Cells were treated with control conditioned media from L cells (Control-CM) or Wnt3A conditioned media from L cells and R-spondin1 conditioned media from HEK293T cells (Wnt3A+Rspo) and luciferase activity from a β-catenin responsive reporter (TOPflash) was measured; p values are indicated on each plot. (**G**) Dual sgRNA strategy to delete the remaining *Fzd2* CDS in the *Fzd2$^{tm1.1Nat}$* allele. The resulting allele is called Fzd2-KO-delta-Cterm. (**H**) *Fzd2$^{KO\text{-}delta\text{-}Cterm}$* E18.5 embryo counts and number of cleft palates identified.

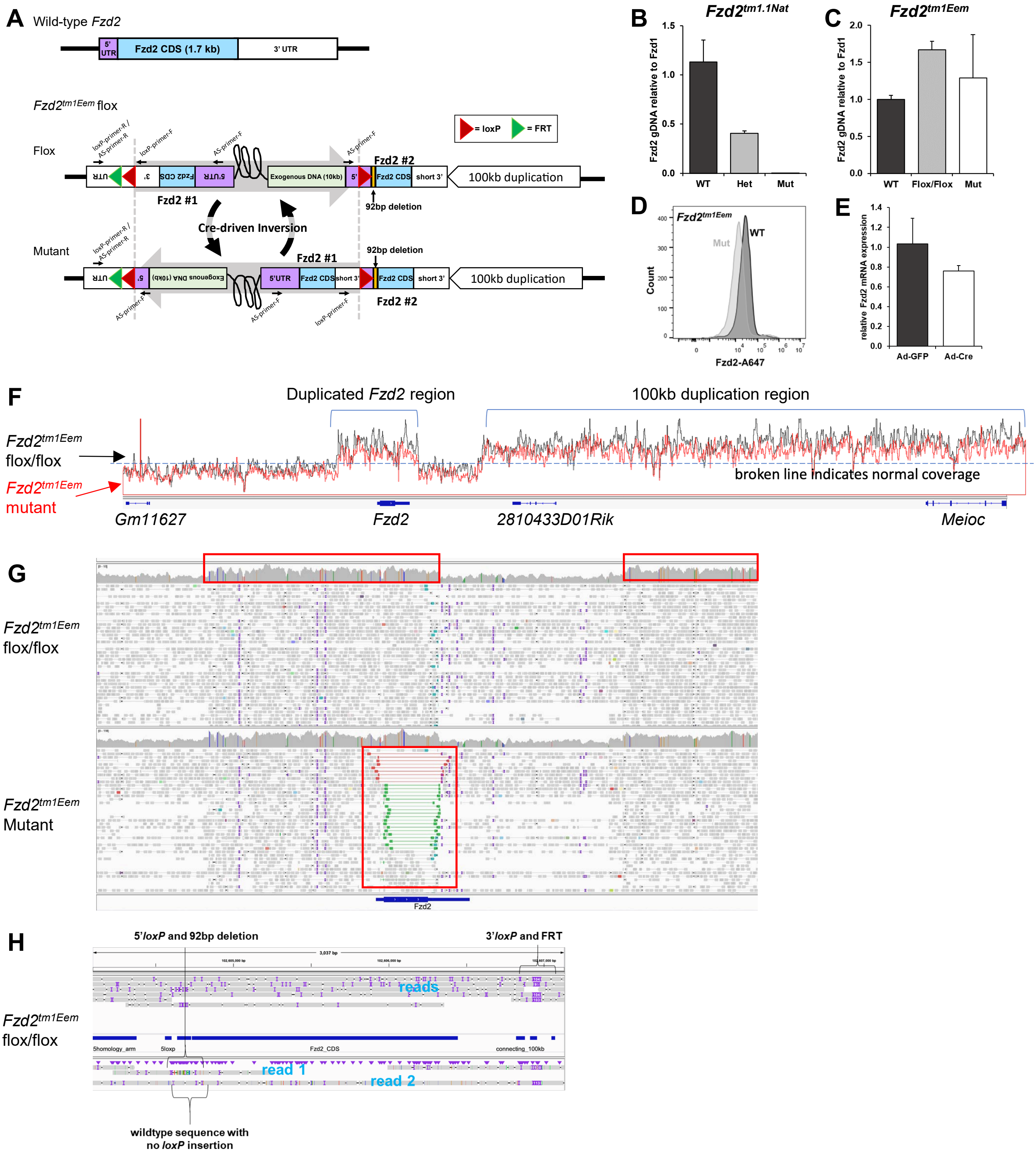


Disease Models & Mechanisms • Supplementary information

**Fig. S2. The published *Fzd2*$^{tm1Eem}$ flox allele is a complex genomic alteration.** (**A**) Maps detailing our working model of the *Fzd2*$^{tm1Eem}$ flox allele. A wild-type Fzd2 gene structure is shown, with 5'UTR in purple, CDS in blue, and 3'UTR in white. The *Fzd2*$^{tm1Eem}$ flox allele contains a duplication of Fzd2, resulting in two copies (blue). There is 10 kb of exogenous DNA (bacterial gDNA and targeting plasmid PL253 sequence, light green) in between the two copies of Fzd2. "Fzd2 #1" contains the 3' *loxP* insertion but not 5'loxP. "Fzd2 #2" contains 5' *loxP* insertion as well as a 92 bp deletion (orange) downstream of the 5' *loxP* insertion site and immediately upstream of the Fzd2 start sequence. "Fzd2 #2" has a shortened 3' UTR that is connected to an inverted 100 kb sequence downstream of the Fzd2 gene (refer to Supplemental Figure 1A for more details). The sequence between the two Fzd2 copies is of unknown length and denoted by a black curved lined (refer to Supplemental Figure 1B for more details). Because we cannot confirm the orientations of the two *loxP* sites within the two Fzd2 copies due to the long sequence between the two Fzd2 copies, we hypothesize cre-driven recombination results in continuous sequence inversion between the two *loxP* sites due to the opposite *loxP* orientations (refer to Discussion for more details). (**B**) Real-time quantitative PCR on genomic DNA shows a 50% decrease in Fzd2 gene content in *Fzd2*$^{tm1.1Nat}$ heterozygotes and no Fzd2 gene content in homozygous mutants (primer targeting the N-terminal region). (**C**) The *Fzd2*$^{tm1Eem}$ display a ~2-fold increase in Fzd2 gene content in flox/flox and ~ homozygous mutant (Mut) samples, which supports Fzd2 being duplicated and *loxP* sites being in opposite orientations. (**D**) FZD2 flow cytometric analysis of E14.5 limb buds from wild-type (WT) or mice homozygous for a germline cre-mediated recombination of the *Fzd2*$^{tm1Eem}$ allele (Mut). Cells were gated to discard debris and doublets, and live cells were determined by DAPI exclusion. FZD2 median fluorescent intensity (MFI) was compared between WT and Mut. WT MFI = 25954; Mut MFI = 12680 (**E**) Limb micromass cultures from homozygous flox *Fzd2*$^{tm1Eem}$ mice were infected with Ad5CMV:eGFP (control) or Ad5CMV:Cre-eGFP at an MOI of 100 and assessed by qRT-PCR for Fzd2 mRNA expression. (**F**) The IGV coverage tracks of *Fzd2*$^{tm1Eem}$ flox/flox and homozygous global mutant WGS reads were merged to show no Fzd2 or other genomic DNA deletion after Cre exposure. (**G**) Illumina sequencing of *Fzd2*$^{tm1Eem}$ (homozygous flox "flox/flox" or homozygous global mutants "Mutant"). Reads were aligned to mm10 mouse reference genome for sequence and structural accuracy. The paired "big insert size" reads in red are possibly caused by Cre-driven inversion indicated in Figure 2A. Note no coverage reduction in the mutant sample. (**H**) Nanopore (ONT) long-read sequencing was performed on gDNA from *Fzd2*$^{tm1Eem}$ flox/flox animals. We identified two kinds of floxed Fzd2 alleles.

Disease Models & Mechanisms • Supplementary information

One has a 5'loxP insertion along with a 92 bp deletion downstream (read 1), while another *Fzd2*$^{tm1Eem}$ allele does not have a 5'loxP or 92 bp deletion but does have a 3'loxP/FRT insertion (read 2 and reads). We do not have a read that contains both 5'loxP insertion and 3'UTR region to confirm the nonexistence of a Fzd2 allele with both 5' and 3' *loxP* insertions, which is inferred by other evidence presented in the Discussion.

Disease Models & Mechanisms • Supplementary information

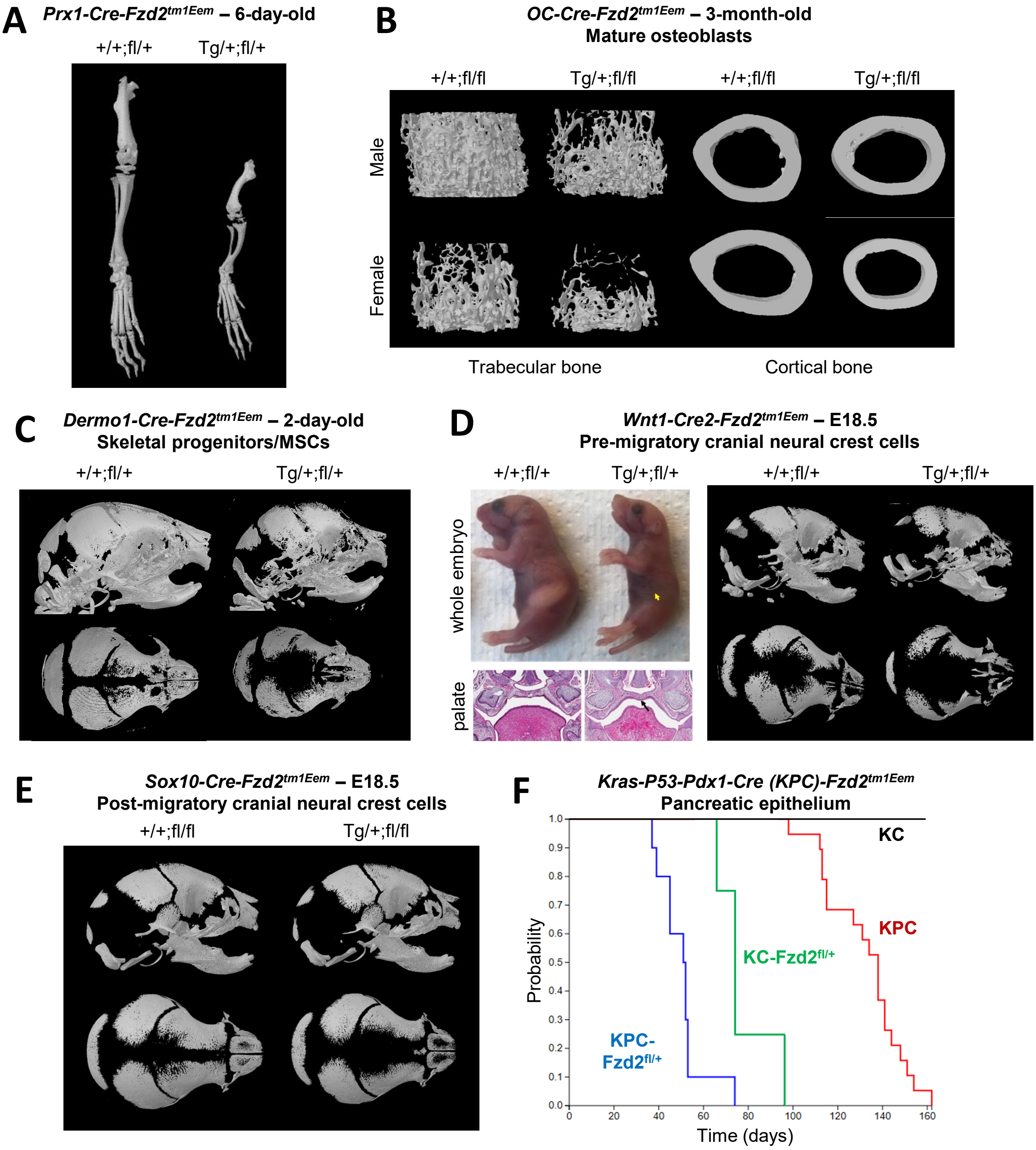


Disease Models & Mechanisms • Supplementary information

**Fig. S3. *Fzd2$^{tm1Eem}$* allele phenotyping.** (**A**) Micro-CT 3D images of 6-day-old hindlimbs from *Prx1-Cre-Fzd2$^{tm1Eem}$* wild-type control (+/+; fl/+) and heterozygous (Tg/+; fl/+) animals. (**B**) Micro-CT 3D image of 3-month-old femurs from *OCN-Cre-Fzd2$^{tm1Eem}$* wild-type control (+/+; fl/fl) and homozygous (Tg/+; fl/fl) animals. Representative images of males and females are shown of distal femoral trabecular bone and midshaft femoral cortical bone. (**C**) Micro-CT 3D images of 2-day-old skulls from *Dermo1-Cre-Fzd2$^{tm1Eem}$* wild-type control (+/+; fl/+) and heterozygous (Tg/+; fl/+) animals. (**D, left**) Gross images of E18.5 whole embryos from *Wnt1-Cre2-Fzd2$^{tm1Eem}$* wild-type control (+/+; fl/+) and heterozygous (Tg/+; fl/+) animals. Yellow arrow indicates lack of milk in stomach in heterozygous pup. H&E images of coronal palatal sections showed closed palates in wild-type control (+/+; fl/+) and heterozygous (Tg/+; fl/+) animals. (**D, right**) Micro-CT 3D images of E18.5 skulls from *Wnt1-Cre2-Fzd2$^{tm1Eem}$* wild-type control (+/+; fl/+) and heterozygous (Tg/+; fl/+) animals. (**E**) Micro-CT 3D images of E18.5 skulls from *Sox10-Cre-Fzd2$^{tm1Eem}$* wild-type control (+/+; fl/fl) and homozygous (Tg/+; fl/fl) animals. (**F**) Kaplan-Meier curve of *Kras-P53-Pdx1-Cre-Fzd2$^{tm1Eem}$* animals. KC = *Kras$^{G12D/+}$;Pdx1-Cre$^{Tg/+}$;Fzd2$^{+/+}$*. KPC = *Kras$^{G12D/+}$;P53$^{R172H/+}$;Pdx1-Cre$^{Tg/+}$;Fzd2$^{+/+}$*. KC-Fzd2$^{fl/+}$ = *Kras$^{G12D/+}$;Pdx1-Cre$^{Tg/+}$;Fzd2$^{fl/+}$*. KPC-Fzd2$^{fl/+}$ = *Kras$^{G12D/+}$;P53$^{R172H/+}$;Pdx1-Cre$^{Tg/+}$;Fzd2$^{fl/+}$*.

Disease Models & Mechanisms • Supplementary information

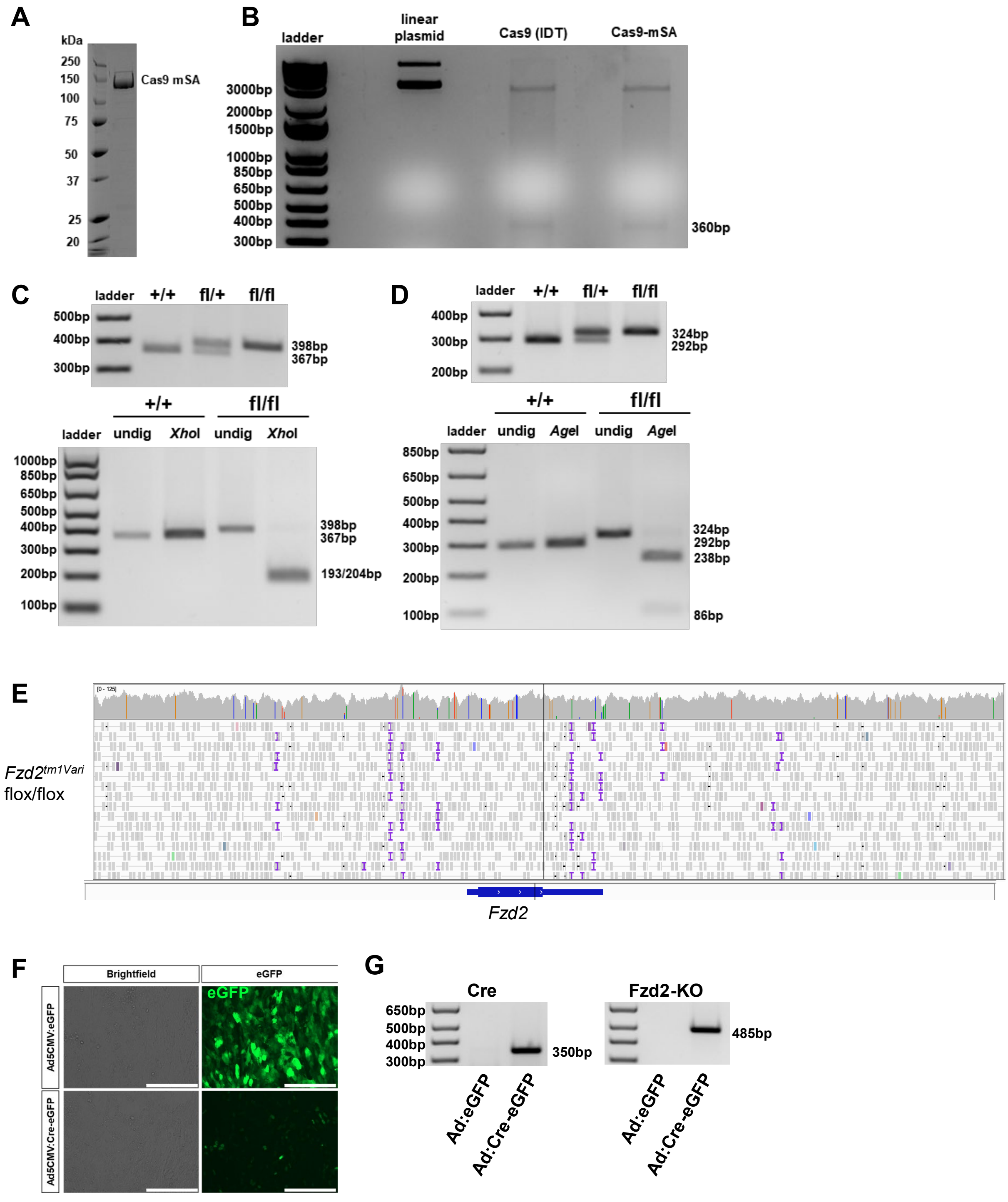


Disease Models & Mechanisms • Supplementary information

**Fig. S4. Confirmation of Cre-mediated recombination of *Fzd2*$^{em1Vari}$ allele.** (**A**) SDS page of Cas9-mSA protein after size exclusion column. (**B**) DNA cleavage activity of Cas9-mSA endonuclease was assayed on linearized plasmid DNA containing eGFP (LentiCMVtight-GFP) and produced a cut product of the expected size (360bp). (**C**) PCR-based genotyping products for 5' *loxP* site (top) and digestion with *Xho*I of flox product (bottom) results in expected products (193 bp and 204 bp). (**D**) PCR-based genotyping products for 3' *loxP* site and digestion with *Age*I of flox product results in the expected products (86 bp and 238 bp). undig = undigested. (**E**) Illumina sequencing of *Fzd2*$^{em1Vari}$ flox/flox allele. Reads were aligned to mm10 mouse reference genome for sequence and structural accuracy. (**F**) Limb micromass cultures from *Fzd2*$^{tmVari}$ flox/flox were infected with Ad5CMV:eGFP (control) or Ad5CMV:Cre-eGFP at an MOI of 100. Brightfield and fluorescent images show infection efficiency. Ad5CMV:eGFP is cytoplasmic and Ad5CMV:Cre-eGFP is nuclear. Scale = 200um. (**G**) PCR-based genotyping for Cre or Fzd2 knockout (KO). Ad5CMV:Cre-eGFP infected cells amplified a product for Cre and Fzd2-KO.

Disease Models & Mechanisms • Supplementary information

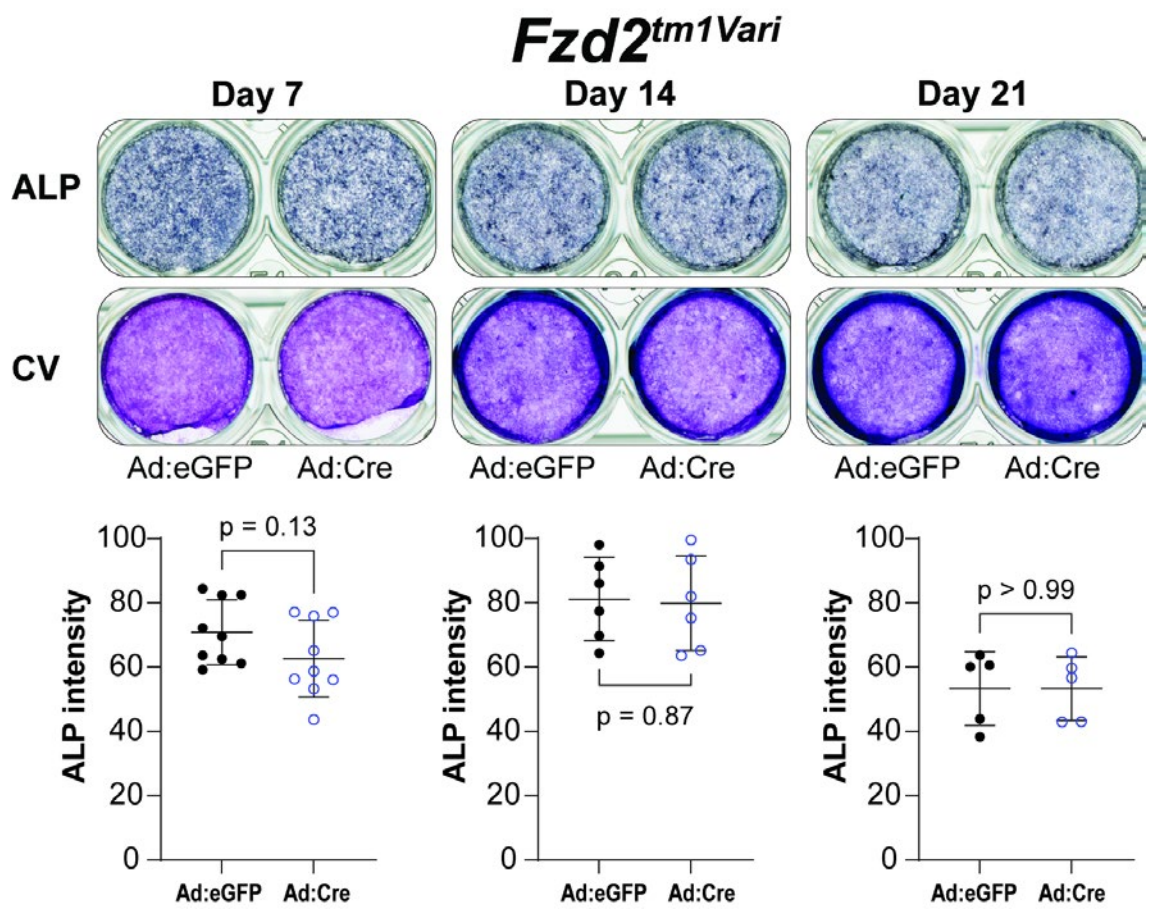


**Fig. S5. Loss of Fzd2 does not impact mineralization *in vitro*.** Calvarial osteoblasts were isolated from 4-day-old *Fzd2*$^{em1Vari}$ pups, cultured, and infected with Ad:eGFP or Ad:Cre-eGFP. Mineralization was induced for 7-21 days. Mineralization was measured by alkaline phosphatase (ALP) staining and cell number was visualized with crystal violet (CV). Representative images for each condition are shown. Relative ALP staining was quantified; individual data points are shown with mean +/- s.d.; p values are indicated on each plot.

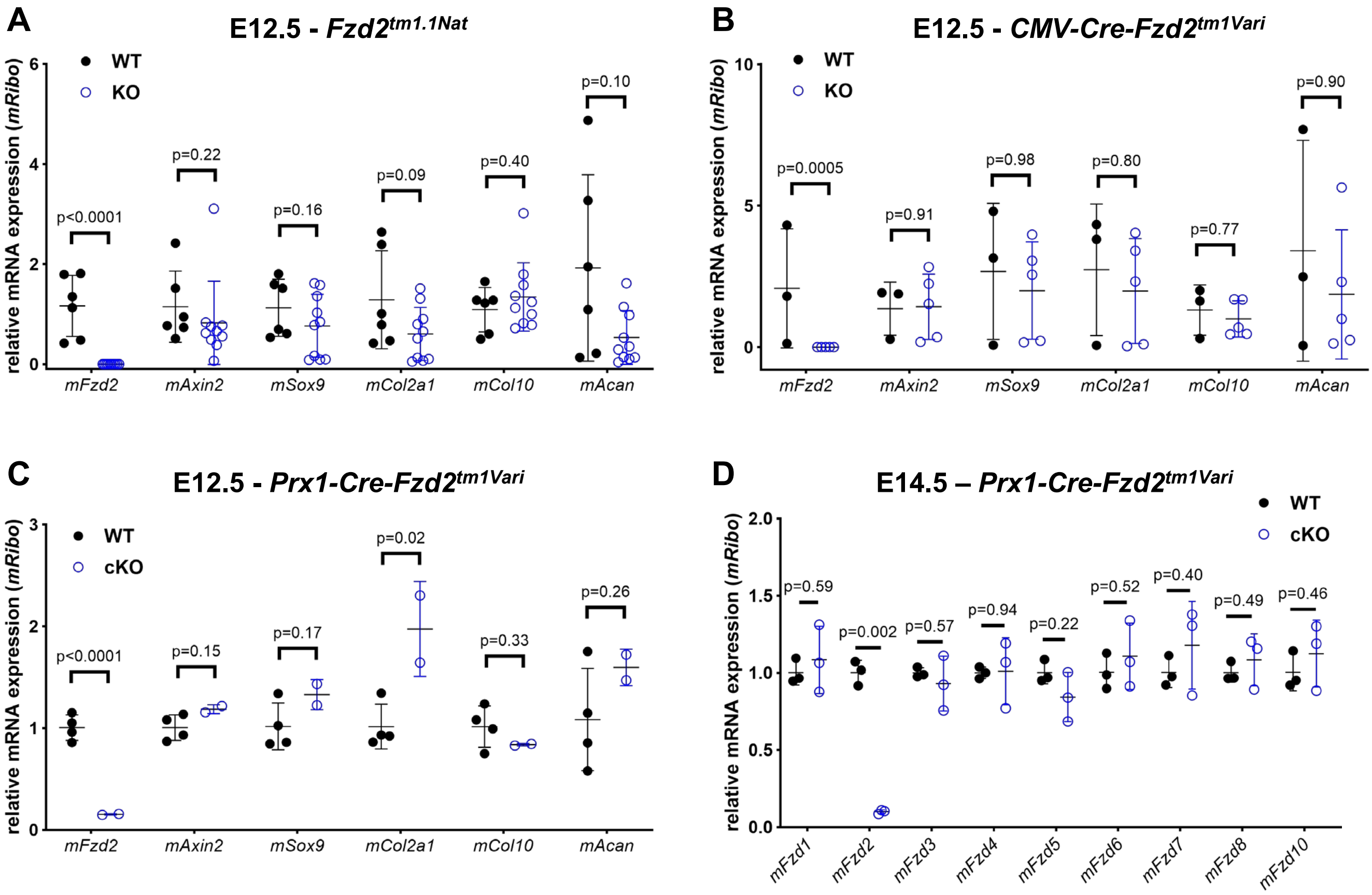


**Fig. S6. Chondrogenic marker expression is largely unchanged at E12.5 across Fzd2 loss-of-function models.** (**A-C**) Quantitative real-time PCR was performed on E12.5 limb digests from *Fzd2*$^{tm1.1Nat}$, *CMV-Cre-Fzd2*$^{em1Vari}$, and *Prx1-Cre-Fzd2*$^{em1Vari}$ animals. cDNA was amplified using primers for *mFzd2, mAxin2, mSox9, mCol2a1, mCol10a1, and mAcan*, and were normalized to *mRibo*. Biological replicates are plotted as individual data points with mean +/- s.d. p values are indicated on each plot. (**D**) Frizzled receptor expression levels were measured in E14.5 limb digests from *Prx1-Cre-Fzd2*$^{em1Vari}$ animals. cDNA was amplified using primers for *mFzd1-8* and *mFzd10*, and were normalized to *mRibo*. Biological replicates are plotted as individual data points with mean +/- s.d. p values are indicated on each plot.

Disease Models & Mechanisms • Supplementary information

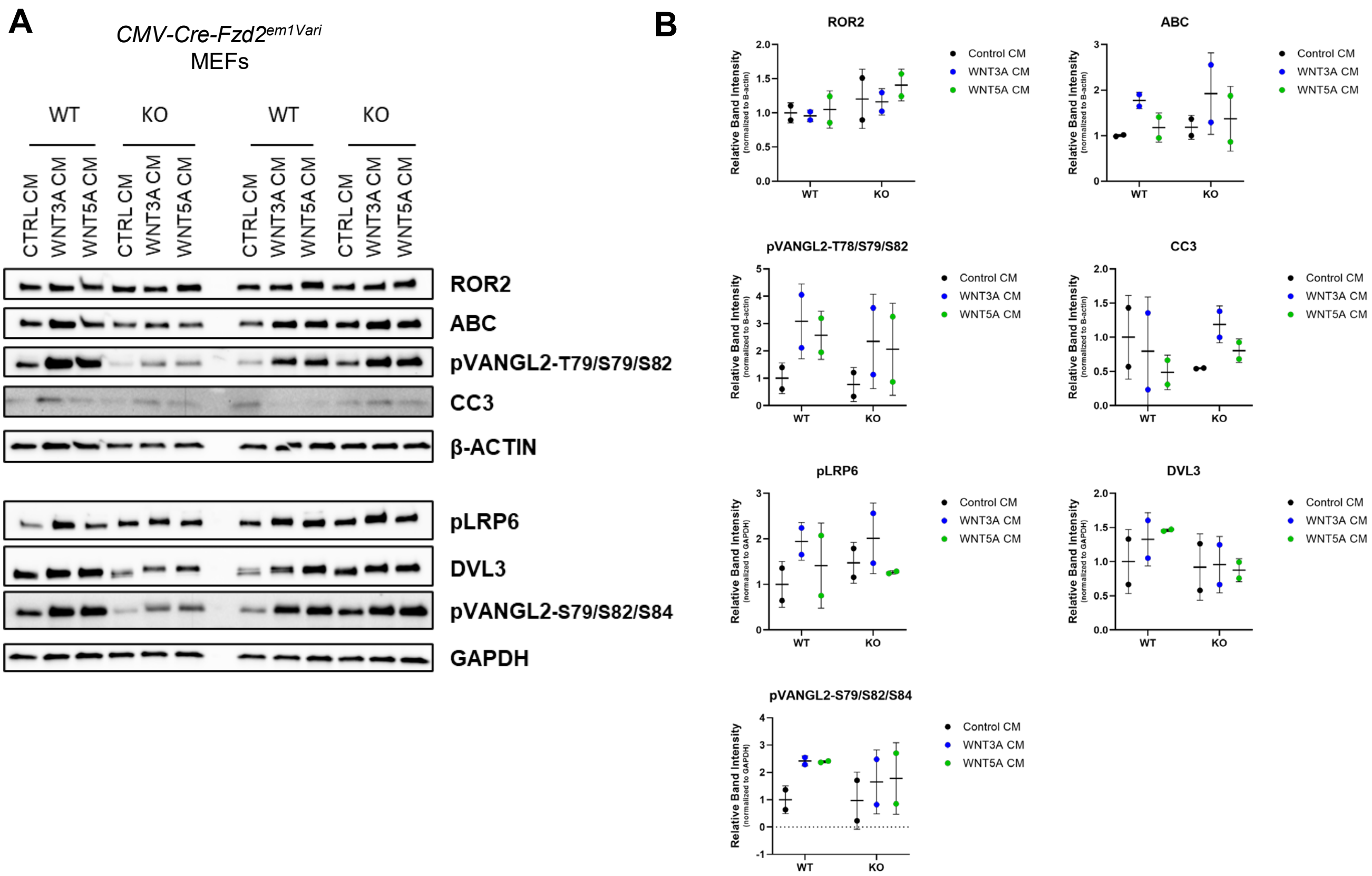


**Fig. S7. Analysis of Wnt pathway and PCP-associated proteins in control and Fzd2-deficient cells.** (**A**) Representative immunoblots from *CMV-Cre-Fzd2*$^{em1Vari}$ MEFs of wild-type (WT) and knockout (KO) genotypes treated with control conditioned media (CTRL CM), WNT3A CM, or WNT5A CM. Blots were probed for ROR2, activated β-catenin (ABC), phosphorylated VANGL2 (pVANGL2-T79/S79/S82), cleaved caspase-3 (CC3), phosphorylated LRP6 (pLRP6), DVL3, and phosphorylated VANGL2 (pVANGL2-S79/S82/S84). β-ACTIN and GAPDH serve as loading controls. (**B**) Quantification of band intensities from immunoblots in (**A**), normalized to loading controls and expressed relative to WT control CM. Data points represent individual biological replicates; mean ± SD is shown.

Disease Models & Mechanisms • Supplementary information

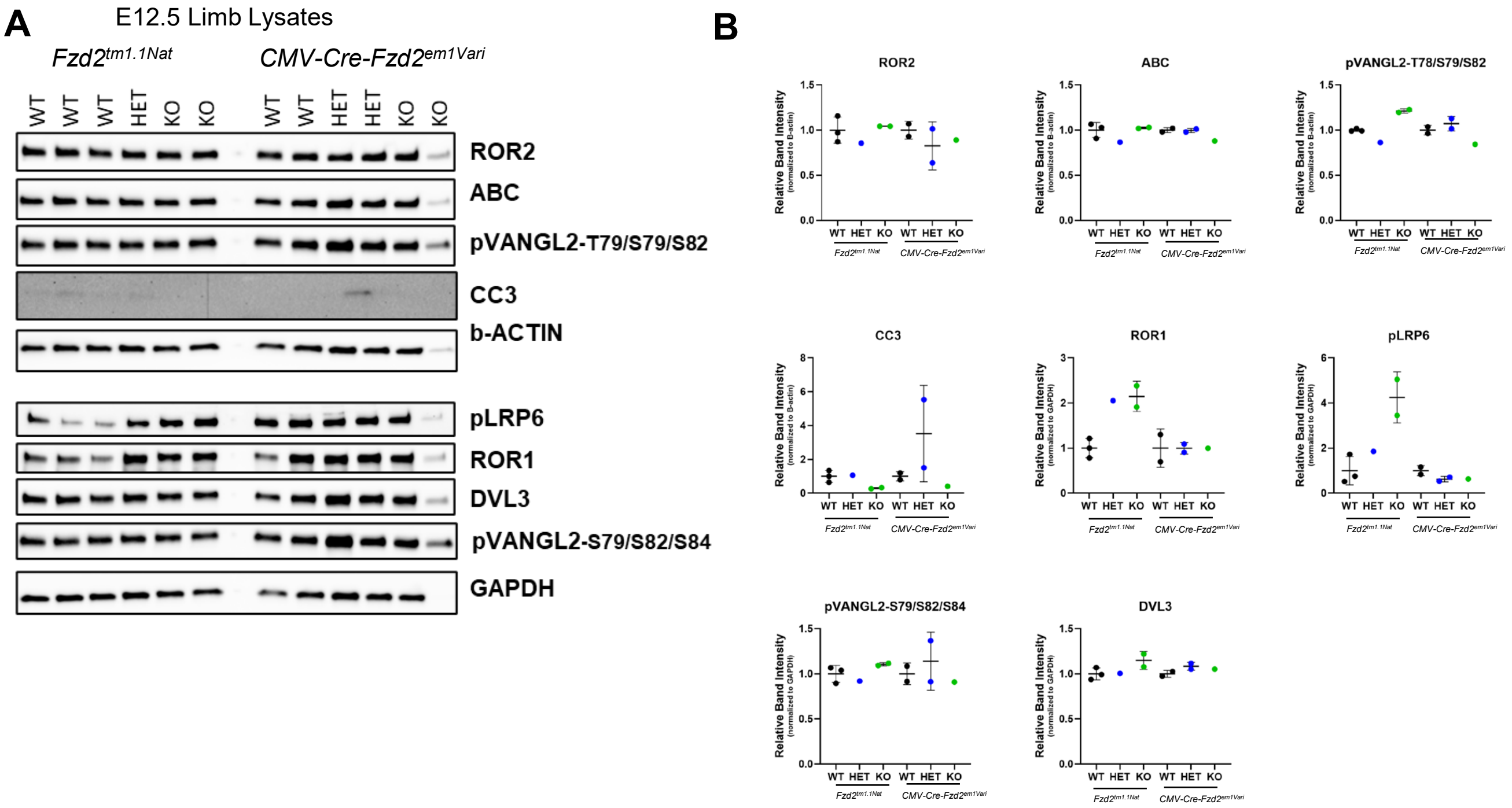


**Fig. S8. Analysis of Wnt pathway and PCP-associated proteins in control and Fzd2-deficient tissues.** (**A**) Representative immunoblots from E12.5 limb lysates of *Fzd2*$^{tm1.1Nat}$ and *CMV-Cre-Fzd2*$^{em1Vari}$ embryos, probed for the same Wnt signaling and PCP-associated proteins as in Fig. S7. WT and KO samples from independent embryos are shown. (**B**) Quantification of band intensities from immunoblots in (**A**), normalized to loading controls and expressed relative to WT control CM. Data points represent individual biological replicates; mean ± SD is shown.

Disease Models & Mechanisms • Supplementary information

**Table S1.** ***Fzd2***$^{tm1.1Nat}$ **adult trabecular and cortical bone micro-CT analyses**

| | Female | | | Male | | |
|---|---|---|---|---|---|---|
| Index | +/+ (N=8) | KO/+ (N=8) | KO/KO (N=8) | +/+ (N=3) | KO/+ (N=5) | KO/KO (N=6) |
| Trab.BMD (μgHA/cm$^3$) | 0.149 ± 0.033 | 0.182 ± 0.040 | 0.160 ± 0.041 | 0.235 ± 0.065 | 0.247 ± 0.056 | 0.225 ± 0.064 |
| Trab.TV (mm$^3$) | 2.783 ± 0.187 | 2.745 ± 0.383 | 2.776 ± 0.180 | 4.050 ± 0.929 | 3.965 ± 0.258 | 3.756 ± 0.729 |
| Trab.BV (mm$^3$) | 0.207 ± 0.093 | 0.273 ± 0.101 | 0.228 ± 0.102 | 0.693 ± 0.369 | 0.720 ± 0.220 | 0.619 ± 0.296 |
| Trab.BV/TV (%) | 7.354 ± 3.004 | 10.077 ± 3.951 | 8.330 ± 4.003 | 16.295 ± 6.752 | 18.088 ± 5.398 | 15.889 ± 6.366 |
| Trab.Th (mm) | 0.050 ± 0.005 | 0.053 ± 0.004 | 0.050 ± 0.005 | 0.056 ± 0.008 | 0.057 ± 0.005 | 0.057 ± 0.010 |
| Trab.Sp (mm) | 0.268 ± 0.027 | 0.250 ± 0.036 | 0.262 ± 0.035 | 0.207 ± 0.019 | 0.202 ± 0.025 | 0.228 ± 0.040 |
| Trab.N (1/mm) | 1.444 ± 0.493 | 1.896 ± 0.707 | 1.625 ± 0.661 | 2.831 ± 0.845 | 3.137 ± 0.780 | 2.713 ± 0.866 |
| Cort.TMD (gHA/cm$^3$) | 1.094 ± 0.017 | 1.106 ± 0.020 | 1.086 ± 0.018 | 1.090 ± 0.040 | 1.071 ± 0.024 | 1.092 ± 0.026 |
| T.Ar (mm$^2$) | 1.526 ± 0.075 | 1.580 ± 0.206 | 1.508 ± 0.310 | 2.081 ± 0.444 | 2.133 ± 0.130 | 1.863 ± 0.259 |
| B.Ar (mm$^2$) | 0.757 ± 0.053 | 0.799 ± 0.101 | 0.778 ± 0.158 | 1.028 ± 0.198 | 1.061 ± 0.081 | 0.971 ± 0.139 |
| CAF | 0.496 ± 0.018 | 0.507 ± 0.033 | 0.517 ± 0.023 | 0.498 ± 0.027 | 0.497 ± 0.013 | 0.521 ± 0.030 |
| Cs.Th (mm) | 0.185 ± 0.011 | 0.193 ± 0.015 | 0.191 ± 0.019 | 0.210 ± 0.006 | 0.217 ± 0.010 | 0.214 ± 0.019 |

**Table S2.** ***Fzd2***$^{em1Vari}$ **adult trabecular and cortical bone micro-CT analyses**

| | Female | | Male | |
|---|---|---|---|---|
| Index | +/+; fl/fl (N=5) | Tg/+; fl/fl (N=6) | +/+; fl/fl (N=8) | Tg/+; fl/fl (N=5) |
| Trab.TV (mm$^3$) | 2.460 ± 0.030 | 2.819 ± 0.297 | 3.179 ± 0.195 | 4.258 ± 0.004** |
| Trab.BV (mm$^3$) | 0.161 ± 0.032 | 0.171 ± 0.061 | 0.639 ± 0.091 | 0.924 ± 0.039* |
| Trab.BV/TV (%) | 6.492 ± 0.622 | 5.960 ± 1.643 | 20.121 ± 2.797 | 21.601 ± 0.474 |
| Trab.Th (mm) | 0.053 ± 0.005 | 0.053 ± 0.002 | 0.074 ± 0.008 | 0.072 ± 0.540 |
| Trab.Sp (mm) | 0.287 ± 0.019 | 0.315 ± 0.024 | 0.218 ± 0.019 | 0.216 ± 0.843 |
| Trab.N (1/mm) | 1.238 ± 0.172 | 1.114 ± 0.299 | 2.736 ± 0.404 | 3.000 ± 0.279 |
| T.Ar (mm$^2$) | 0.735 ± 0.062 | 0.786 ± 0.036 | 1.002 ± 0.075 | 1.201 ± 0.100** |
| B.Ar (mm$^2$) | 0.655 ± 0.054 | 0.702 ± 0.034 | 0.899 ± 0.065 | 1.077 ± 0.091** |
| CAF (%) | 0.891 ± 0.002 | 0.892 ± 0.006 | 0.897 ± 0.005 | 0.896 ± 0.003 |
| Cs.Th (mm) | 0.165 ± 0.004 | 0.169 ± 0.010 | 0.195 ± 0.008 | 0.201 ± 0.011 |

* $P$<0.05, ** $P$<0.01

Disease Models & Mechanisms • Supplementary information
